# FOXC1 regulates endothelial CD98 (LAT1/4F2hc) expression in retinal angiogenesis and blood-retina barrier formation

**DOI:** 10.1038/s41467-024-48134-2

**Published:** 2024-05-16

**Authors:** Teena Bhakuni, Pieter R. Norden, Naoto Ujiie, Can Tan, Sun Kyong Lee, Thomas Tedeschi, Yi-Wen Hsieh, Ying Wang, Ting Liu, Amani A. Fawzi, Tsutomu Kume

**Affiliations:** 1 Department of Medicine, Feinberg Cardiovascular and Renal Research Institute, Feinberg School of Medicine, Northwestern University, Chicago, IL USA; 2 Department of Ophthalmology, Northwestern University Feinberg School of Medicine, Chicago, IL USA; 3 Department of Cardiovascular Medicine, Mayo Clinic, Rochester, MN USA

**Keywords:** Angiogenesis, Experimental models of disease, TOR signalling

## Abstract

Angiogenesis, the growth of new blood vessels from pre-existing vasculature, is essential for the development of new organ systems, but transcriptional control of angiogenesis remains incompletely understood. Here we show that FOXC1 is essential for retinal angiogenesis. Endothelial cell (EC)-specific loss of *Foxc1* impairs retinal vascular growth and expression of *Slc3a2* and *Slc7a5*, which encode the heterodimeric CD98 (LAT1/4F2hc) amino acid transporter and regulate the intracellular transport of essential amino acids and activation of the mammalian target of rapamycin (mTOR). EC-*Foxc1* deficiency diminishes mTOR activity, while administration of the mTOR agonist MHY-1485 rescues perturbed retinal angiogenesis. EC-*Foxc1* expression is required for retinal revascularization and resolution of neovascular tufts in a model of oxygen-induced retinopathy. *Foxc1* is also indispensable for pericytes, a critical component of the blood-retina barrier during retinal angiogenesis. Our findings establish FOXC1 as a crucial regulator of retinal vessels and identify therapeutic targets for treating retinal vascular disease.

## Abstract (web summary)

FOXC1 transcription factor plays a role in vascular development, but its mechanism is not well defined. Here, the authors show FOXC1 regulates retinal angiogenesis by promoting CD98 amino acid transporter expression to activate mTOR signalling, and also by promoting blood-retina barrier formation.

## Introduction

The growth of new vessels from the pre-existing vasculature, termed angiogenesis, is critical during development to support the growth of new organ systems including the retina. This process is also involved in several postnatal physiological mechanisms, such as tissue repair and wound healing. In contrast, dysregulation of neovascularization contributes to the progression of several infectious, inflammatory, malignant, and ischemic disorders, such as retinopathies^1^. Molecular regulation of angiogenesis is controlled by numerous molecular mechanisms including fibroblast growth factor (FGF), vascular endothelial growth factor (VEGF), and Tie receptor tyrosine kinases^2^, the Phosphoinositide 3-kinase (PI3K)/AKT/mammalian target of rapamycin (mTOR) signaling pathway^3^, and cellular metabolism^4^. Tightly coordinated regulation of these mechanisms is necessary for the development of tissue-specific endothelial function and heterogeneity^5^. While significant progress has been made in characterizing the molecular mechanisms contributing to tissue-specific EC function and heterogeneity, our understanding of the angiogenic processes regulating diverse endothelial functions remains incomplete. Particularly, the roles of the transcription factors that may regulate EC gene expression during these processes have yet to be fully elucidated.

FOXC1 and FOXC2 are closely related members of the FOX transcription factor family and share an overlapping expression pattern in the cardiovascular system^6^. Work from our laboratory and others has demonstrated that global knockout mouse models of either FOXC1, FOXC2, or both FOXCs are phenotypically characterized by numerous vascular developmental defects^7–9^. Of clinical significance, mutations in *FOXC1* are predominately associated with Axenfeld-Rieger syndrome, which is characterized by abnormal anterior eye segment dysgenesis and secondary glaucoma in a subset of patients^10^. A cohort of Axenfeld-Rieger Syndrome (ARS) patients also exhibited cerebral small vessel disease (CSVD) as a result of abnormal FOXC1 function, thus implicating FOXC1 dysfunction in the progression of CSVD and stroke^11^. In contrast, mutations in *FOXC2* are predominately associated with hereditary lymphedema-distichiasis syndrome and perturbed lymphatic function^12^.

In humans, the retina begins to vascularize in utero at approximately 16 weeks of gestational age and humans are born with fully developed retina vasculature and regressed hyaloid vessels. In contrast, mice have immature retinal vasculature and persistent hyaloid vessels at birth, with progression of retinal vascular angiogenesis and regression of the hyaloid vessels occurring entirely postnatally, completed by the end of the third postnatal week^13^. The retina vasculature originates from the optic nerve head and the superficial vascular plexus begins to grow towards the periphery of the retina cup to supply hypoxic regions, followed by sprouting into the neuronal layers of the retina to assemble into mature deep and intermediate vascular plexuses^13^. The murine retina vasculature has been studied extensively to characterize the transcriptional regulation of sprouting angiogenesis^14^. However, the direct role of EC-FOXC1/C2 during sprouting angiogenesis remains unknown.

Retinal ischemia characterizes a group of retinopathies, including diabetic retinopathy, a major cause of acquired blindness in the working-age population, and retinopathy of prematurity (ROP), a major cause of acquired blindness in children. The pathogenesis of these retinopathies is associated with pathological retinal neovascularization^13^. While key differences between human and murine retinal developmental angiogenesis exist, the murine oxygen-induced retinopathy (OIR) model has become a crucial tool for investigating molecular mechanisms associated with the pathogenesis of ROP and has aided in the development of several therapeutic strategies^15^. During phase 1 of OIR, mice are placed in hyperoxic conditions (75% oxygen) from postnatal day (P)7 – P12, which mimics the relative hyperoxia premature infants are exposed to at birth. Here, hyperoxic conditions suppress VEGF expression, which induces vessel regression and vaso-oblieration in the central retina and impairs the physiological development of the retinal vasculature. Transfer of mice back to room air at P12 results in relative hypoxia of the vaso-obliterated regions, which in turn induces the expression of pro-angiogenic factors. Induction of pro-angiogenic factors then promotes competing processes of both physiological revascularization into areas of vaso-obliteration and pathological angiogenesis at the border of avascular and vascularized regions, resulting in the formation of leaky, small-caliber neovascular tufts, defining phase 2 of OIR^15^. Current therapies for ischemic retinopathies, such as ROP, have primarily focused on anti-VEGF treatment to address pathological neovascularization. However, a subset of 15−40% of eyes fail to respond or only partially respond to this therapy^16^, and there is the potential risk of neurodevelopmental delays in infants^17^. These limitations underscore a need for novel molecular targets and alternative therapeutic strategies.

Here, we show that EC-FOXC1 transcriptional activity, and not EC-FOXC2, is necessary for physiological retinal angiogenesis during postnatal development in mice. Inducible, endothelial cell (EC)-specific deletion of *Foxc1*, *Foxc2*, or both factors shows that loss of EC-*Foxc1* results in impaired angiogenesis, while deletion of *Foxc2* resulted in minimal changes. Additionally, compound deletion of *Foxc1* and *Foxc2* does not result in an additive effect, thus underscoring the importance of *Foxc1*. RNA-sequencing analysis of isolated retinal ECs shows that *Foxc1* mRNA is more highly expressed than *Foxc2*, demonstrating a predominant role for FOXC1 in transcriptional regulation, and identified differentially downregulated genes, including two solute carrier (SLC) family members associated with regulation of amino acid transport and mTOR signaling activation, *Slc3a2* (encoding the 4F2 cell-surface antigen heavy chain, 4F2hc) and *Slc7a5* (encoding L-type amino acid transporter 1, LAT1). We show that FOXC1 is capable of directly binding to transcriptionally active regions of the *SLC3A2* and *SLC7A5* loci in cultured ECs and that *FOXC1* knockdown results in a reduction in their expression levels. Consistently, mTOR signaling activity is significantly reduced in EC-specific *Foxc1* knockout mice, whereas stimulation of mTOR signaling with the chemical agonist MHY-1485 could partially rescue the impaired angiogenesis phenotype. Furthermore, we show that FOXC1 is necessary for physiological revascularization and proper formation of the blood-retina barrier (BRB) of the retina during OIR. We also reveal that pericyte-specific *Foxc1* deletion impairs the BRB during postnatal angiogenesis. Collectively, our data demonstrates a critical role for FOXC1 in transcriptional regulation of retinal angiogenesis both during development and in ischemic retinopathies and suggests new targets for therapeutic intervention addressing FOXC1-associated vascular dysfunction.

## Results

### FOXC1 is required for retina angiogenesis in postnatal development

**Fig. 1:**
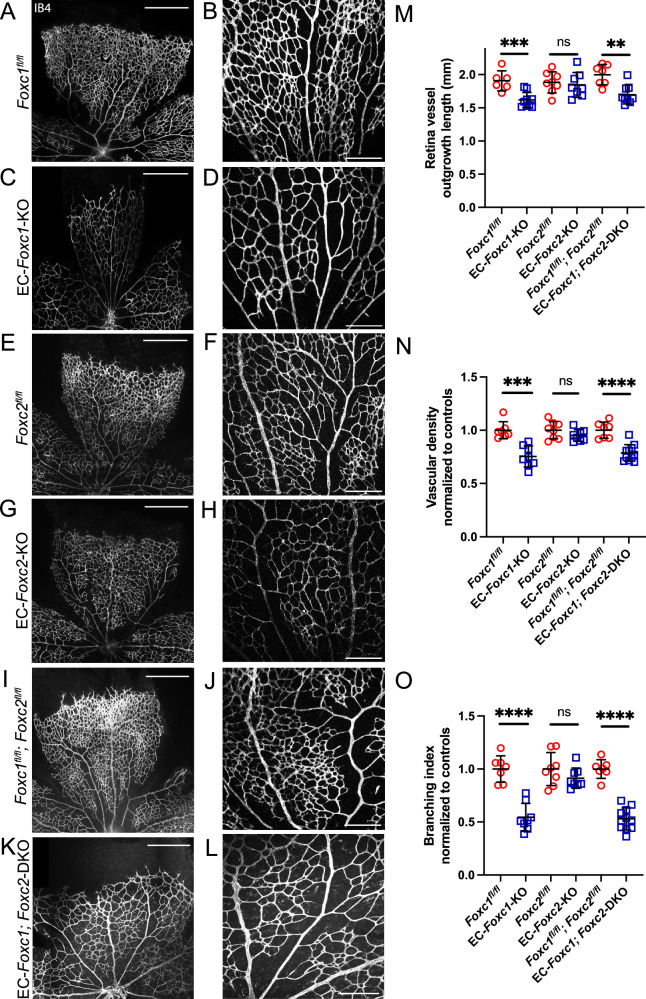
Endothelial *Foxc1* is required for postnatal retina angiogenesis. Representative images of isolectin-B4 (IB4) staining of retina leaflets and the rear capillary plexus in separate or same fields of different individuals in P6 littermate control (**A**, **B**, **E**, **F**, **I**, **J**) or EC-*Foxc1*-KO (**C**, **D**), EC-*Foxc2*-KO (**G**, **H**), or EC-*Foxc1; Foxc2*-DKO (**K**, **L**) mice. Scale bars are 500 and 200 μm respectively. **M** Quantification of retina vessel outgrowth length in P6 EC-*Foxc1*-KO (*n* = 9), EC-*Foxc2*-KO (*n* = 8), or EC-*Foxc1; Foxc2*-DKO (*n* = 9) mice compared to the *Foxc1*^*fl/fl*^ (*n* = 6), *Foxc2*^*fl/fl*^ (*n* = 8) and *Foxc1*^*fl/fl*^*; Foxc2*^*fl/fl*^ (*n* = 7) mice. Data are mean ± SD. ns, not significant; **p = 0.0011; ***p = 0.0010. Student’s two-tailed, unpaired *t* test. Quantification of vascular density per 10X HPF (**N**), and branching index per 10X HPF (**O**) in P6 EC-*Foxc1*-KO (*n* = 8), EC-*Foxc2*-KO (*n* = 8), or EC-*Foxc1; Foxc2*-DKO (*n* = 11) mice compared to the *Foxc1*^*fl/fl*^ (*n* = 7), *Foxc2*^*fl/fl*^ (*n* = 8) and *Foxc1*^*fl/fl*^*; Foxc2*^*fl/fl*^ (*n* = 7) mice. Data are mean ± SD. ns, not significant; ***p = 0.0002; ****p < 0.0001. Student’s two-tailed, unpaired *t* test. Source data are provided as a Source Data file.

To investigate the role of FOXC1/FOXC2 in transcriptional regulation of angiogenesis, we performed tamoxifen administration from postnatal day (P)1 to P5 in endothelial cell (EC)-specific *Foxc* knockout mouse lines^18^ and assessed the retinal vasculature at P6 (Fig. 1). EC-*Foxc1*-KO (*Cdh5-Cre*^*ERT2*^*; Foxc1*^*fl/fl*^) mice exhibited impaired retina angiogenesis associated with significantly reduced radial outgrowth of retinal vessels and reduced vascular density and branching within the capillary plexus (Fig. 1A–D, M–O). In contrast, retinal vessel outgrowth, capillary plexus vascular density, and branching in EC-*Foxc2*-KO mice (*Cdh5-Cre*^*ERT2*^*; Foxc2*^*fl/fl*^) showed no significant differences (Fig. 1E–H, M–O), yet these mice exhibit severe lymphatic vascular dysfunction in the mesenteric collecting vessels at P6^18^. We previously reported that compound EC-specific deletion of *Foxc1* and *Foxc2* (*Cdh5-Cre*^*ERT2*^*; Foxc1*^*fl/fl*^*; Foxc2*^*fl/fl*^, referred to as EC-*Foxc1; Foxc2*-DKO) resulted in an additive effect where lymphatic valves were nearly ablated within the mesentery lymphatic collecting vessels^18^. EC-*Foxc1; Foxc2*-DKO mice exhibited impaired retina angiogenesis (Fig. 1I–O). Some EC-*Foxc1; Foxc2*-DKO mice had crossings of arterioles and venules known as arteriovenous nicking (5/21 mice, 23.8%), and two of these mutants exhibited double arterial crossings. However, no additive effect was observed when compared to phenotypes in EC-*Foxc1*-KO mice (Fig. 1M–O), suggesting that FOXC1 primarily regulates retina angiogenesis compared to FOXC2.

**Fig. 2:**
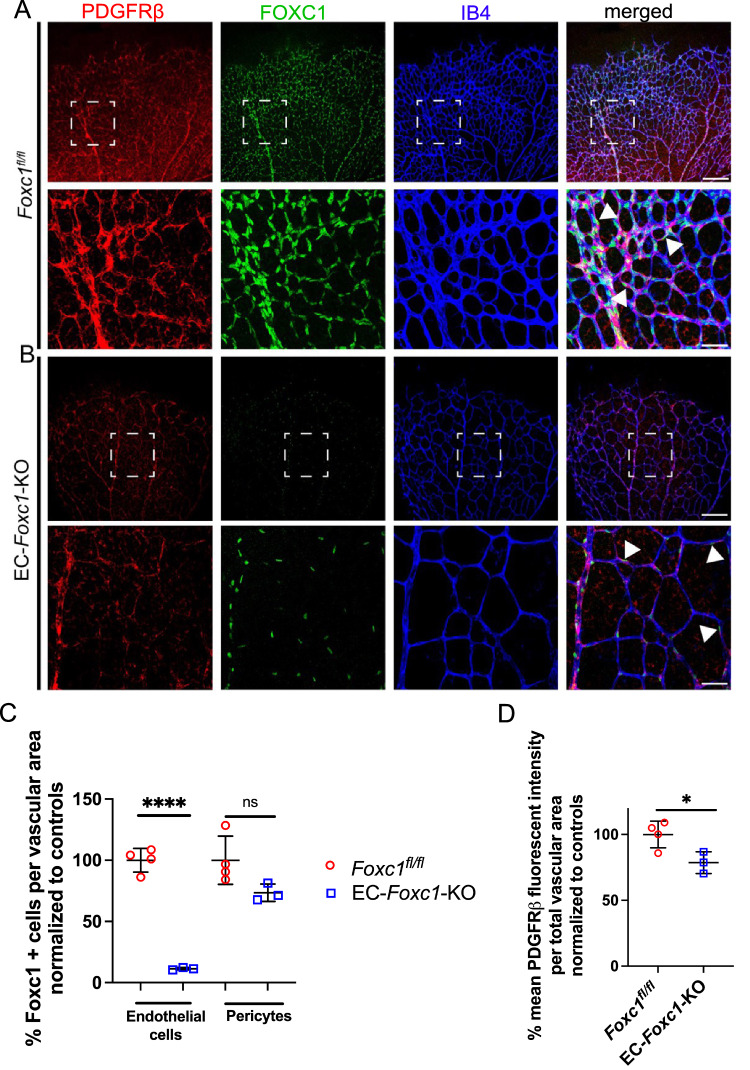
FOXC1 is expressed globally throughout the retina vasculature in both endothelium and pericytes. Representative images of PDGFRβ, FOXC1, and IB4 staining in the retina vasculature of P6 control (**A**) and EC-*Foxc1*-KO (**B**) individuals. White boxes denote magnified regions depicted in lower panels. White arrowheads denote FOXC1+ and PDGFRβ+ pericytes recruited to the abluminal surface of the retina endothelium in both control and EC-*Foxc1*-KO mutant mice. Scale bars are 200 µm and 50 µm respectively. Quantification of FOXC1-positive cells in the endothelial cells and pericytes per vascular area (**C**) and % mean PDGFRβ fluorescent intensity per total vascular area (**D**) in P6 EC-*Foxc1*-KO (*n* = 3) and littermate controls (*n* = 4). Data are mean ± SD. ns, not significant; *p = 0.0312, ****p < 0.0001. Two-tailed unpaired *t* test. Source data are provided as a Source Data file.

FOXC1 expression in the retinal vasculature at P6 showed broad expression within the nuclei throughout capillaries, arterioles, venules, and PDGFRβ + pericytes (Fig. 2A). EC-*Foxc1*-KO mice showed a strong reduction of nuclear FOXC1 expression within the retinal endothelium, however, PDGFRβ/FOXC1 + pericytes were still localized to the abluminal surface of vessels, suggesting that endothelial-specific deletion of FOXC1 does not appear to impair pericyte recruitment (Fig. 2B, C). Interestingly, the fluorescent intensity of PDGFRβ staining was significantly decreased in EC-*Foxc1*-KO retinas (Fig. 2D), indicating a defect in EC-pericyte communication, caused by the lack of *Foxc1* in retinal ECs. Attempts to characterize FOXC2 expression by immunostaining proved difficult, suggesting that FOXC1 expression is enriched within the retinal endothelium compared to FOXC2. Supporting this, RNA-sequencing analysis from ECs isolated from pooled P6 retinas (discussed in further detail in subsequent sections) revealed that *Foxc1* expression is over 100-fold greater than *Foxc2* in the neonatal retina vasculature of control mice (Supplementary Data 1), thus supporting our observations for a predominant role of FOXC1 in retinal angiogenesis.

To further characterize phenotypes associated with *Foxc* deletion in the retina vasculature, we analyzed vascular density and branching at the arterioles and venules of the angiogenic front (Supplementary Fig. 1). EC-*Foxc1*-KO mice exhibited significantly reduced vascular density and branching at both the arteriole (Supplementary Fig. 1A, B, G, H) and venule fronts (Supplementary Fig. 1I, J, O, P) whereas EC-*Foxc2*-KO mice only showed a modest, yet statistically significant, reduction in branching at the arteriole front (Supplementary Fig. 1C, D, G, H, K, L, O, P). Similarly, the combined deletion of *Foxc1* and *Foxc2* resulted in significantly reduced vascular density and branching at both fronts, but no additive effects of the loss of the two *Foxc* genes were observed (Supplementary Fig. 1E–H, M–P). Cytoskeletal filopodia extensions enriched with VEGFR-2 mediate tip cell migration towards VEGF-A gradients during sprouting angiogenesis^19^. We also assessed whether filopodia number along the angiogenic front was impacted by loss of endothelial *Foxc* expression and observed that the total number of filopodia was significantly reduced in EC-*Foxc1*-KO and EC-*Foxc1; Foxc2*-DKO mice, but not EC-*Foxc2*-KO mice, indicating that sprouting angiogenesis is impaired as a result of loss of FOXC1 function (Supplementary Fig. 2A–G).

**Fig. 3:**
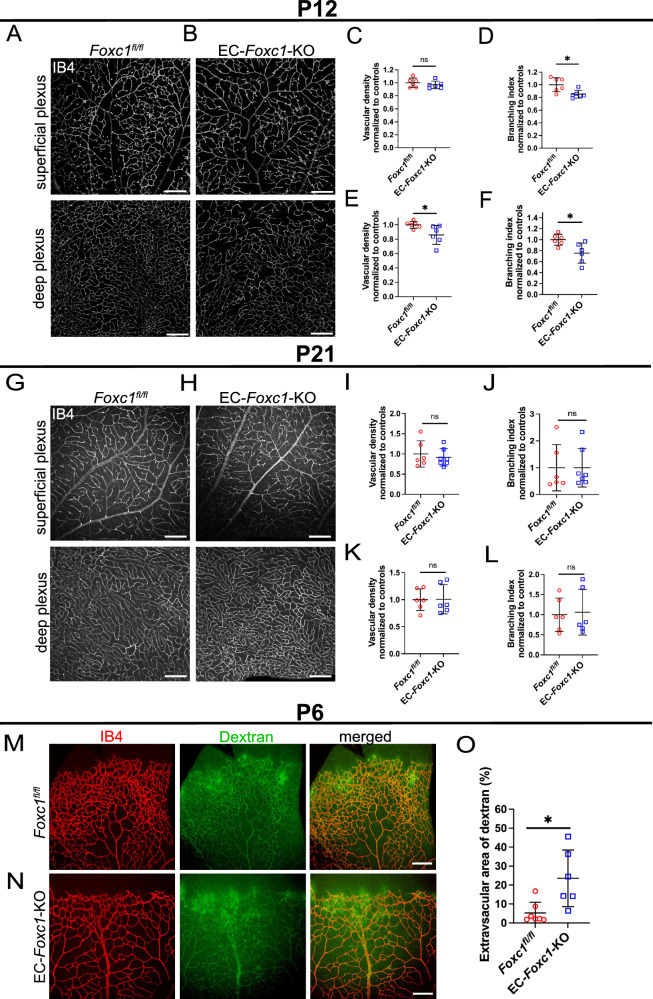
Early postnatal endothelial-specific deletion of *Foxc1* impairs deep plexus formation of the retina vasculature. Representative images of IB4 staining in the superficial and deep vascular plexus of P12 littermate control (**A**) and EC-*Foxc1*-KO (**B**) individuals. Scale bars are 200 µm. Quantification of vascular density per 10X HPF (**C**) and branching index per 10X HPF (**D**) in the superficial vascular plexus of P12 EC-*Foxc1*-KO (*n* = 6) mice compared to littermate controls (*n* = 6). Data are mean ± SD. ns, not significant; *p = 0.0169. Two-tailed unpaired *t* test. Quantification of vascular density per 10X HPF (**E**) and branching index per 10X HPF (**F**) in the deep vascular plexus of P12 EC-*Foxc1*-KO (*n* = 6) mice compared to littermate controls (*n* = 6). Data are mean ± SD. ns, not significant; *p < 0.05. Two-tailed unpaired *t* test. Representative images of IB4 staining in the superficial and deep vascular plexus of P21 littermate control (**G**) and EC-*Foxc1*-KO (**H**) mice. Scale bars are 200 μm. Quantification of vascular density per 10X HPF (**I**) and branching index per 10X HPF (**J**) in the superficial vascular plexus of P21 EC-*Foxc1*-KO (*n* = 7) mice compared to littermate controls (*n* = 6). Quantification of vascular density per 10X HPF (**K**) and branching index per 10X HPF (**L**) in the deep vascular plexus of P21 EC-*Foxc1*-KO mice (*n* = 6) compared to littermate controls (*n* = 6). Data are mean ± SD. ns, not significant. Two-tailed unpaired *t* test. **M**, **N** Representative images of retina vessel leakage assessed by retro orbital injections of FITC-dextran and IB4-568 in P6 littermate control (**M**) and EC-*Foxc1*-KO (**N**) individuals. Scale bars are 200 µm. **O** Quantification of extravascular dextran leakage in the retinas of EC-*Foxc1*-KO (*n* = 6) and littermate controls (*n* = 7) mice at P6. The dextran positive area subtracted from the IB4 positive area is regarded as the extravascular dextran leakage area and has been presented as %. Data are mean ± SD. *p = 0.0119. Two-tailed unpaired *t* test. Source data are provided as a Source Data file.

To characterize the impaired angiogenesis and vascular growth deficiency phenotype observed in EC-*Foxc1*-KO mice, we performed the analysis of endothelial proliferation by quantification of phospho-histone H3+ ECs during postnatal angiogenesis. Endothelial proliferation was significantly reduced in EC-*Foxc1*-KO mice (Supplementary Fig. 3A–C). However, analysis of apoptosis via quantification of cleaved caspase 3+ ECs showed that there was a trend towards increased EC death in EC-*Foxc1*-KO mice, although the difference was not statistically significant (Supplementary Fig. 3D–F). Thus, impaired retinal vascular development in EC-*Foxc1*-KO mice is primarily associated with reduced EC proliferation. To assess whether impairments in retinal angiogenesis could be resolved as development progresses, we administered tamoxifen from P1 to P5 and analyzed retinas at P12 (Fig. 3A–F). Analysis of the superficial retina vascular plexus showed that vascular density was comparable to littermate controls, but that branching was modestly, yet significantly, reduced indicative of partial recovery of vascular development within the retinas of EC-*Foxc1*-KO mice (Fig. 3A–D). In contrast, analysis of the deep plexus at P12 showed that vascular density and branching were significantly reduced in EC-*Foxc1*-KO mice (Fig. 3E, F), demonstrating that early loss of *Foxc1* impairs vascular sprouting both during early retina vascular outgrowth and sprouting into the retina tissue during formation of the deep plexus. We further examined the superficial and deep vascular plexus of P21 retinas of control and EC-*Foxc1*-KO mice, and there was no significant difference in both superficial and deep plexus layers between control and EC-*Foxc1*-mutant mice (Fig. 3G–L). This observation suggests that retinal vascularization in EC-*Foxc1*-KO mice is initially defective but reaches normal at later stages. Since the retinal vessels of EC-*Foxc1*-KO mice showed a defect in pericytes (Fig. 2D), retina vessel leakage was assessed by retro-orbital injections of FITC-dextran and IB4-568. We found that *Foxc1*-mutant retinal vessels at P6 were much leakier than the controls (Fig. 3M–O), whereas P21 EC-*Foxc1*-mutant retinal vessels appeared functional (i.e., non-leaky vessels) as seen in the controls (Supplementary Fig. 4).

In summary, endothelial FOXC1 deficiency leads to impaired angiogenesis associated with reduced vascular outgrowth and endothelial proliferation, yet endothelial FOXC2 deficiency does not strongly affect the retinal vasculature. Thus, FOXC1 has a key role in regulating blood vascular growth during postnatal retina development whereas FOXC2 primarily regulates the growth and maintenance of the lymphatic vasculature during postnatal development^18,20^.

### FOXC1 regulates CD98 expression in the retina vasculature

**Fig. 4:**
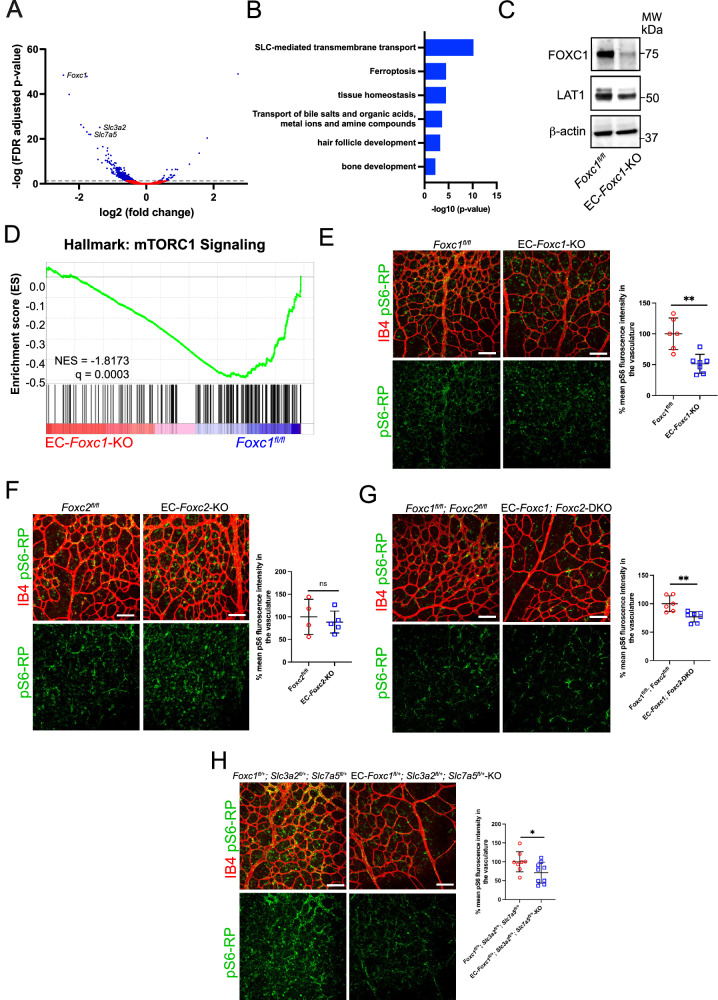
FOXC1 deficiency in the retina vasculature reduces expression of *Slc3a2/Slc7a5* and activation of mTOR signaling during angiogenesis. **A** Volcano plot showing DEGs between EC-*Foxc1*-KO mice and littermate controls in retina vasculature of P6 mice. Dashed line denotes statistical significance cutoff from FDR-adjusted *p* < 0.05 and adjusted for multiple testing using Benjamini and Hochberg approach. **B** Bar plots of subsets of GO gene sets over-represented among genes downregulated in EC-*Foxc1*-KO mice with log2 fold change ≤ −1 compared to littermate controls. X-axis values are represented as −log10(*p* value) of each associated GO gene set identified from list of DEGs with -log2 fold change and FDR-adjusted *p* < 0.05 inputted into Metascape^65^. **C** Western blot of FOXC1, LAT1 and β-actin from pooled whole-retina protein lysates from P6 EC-*Foxc1*-KO mice and littermate controls. **D** GSEA showing significantly enriched mTORC1 signaling gene set in *Foxc1*^*fl/fl*^ group compared to EC-*Foxc1*-KO mice. **E** Representative images of IB4 and phosphorylated S6 ribosomal protein (pS6-RP) staining of retina vasculature in P6 littermate control and EC-*Foxc1*-KO individuals. Mean pS6-RP intensity quantification in vasculature of EC-*Foxc1*-KO mice (*n* = 7) compared to littermate controls (*n* = 6). Data are mean ± SD. **p = 0.0014. Two-tailed unpaired *t* test. **F** Representative images of IB4 and pS6-RP staining of retina vasculature in P6 littermate control and EC-*Foxc2*-KO individuals. Mean pS6-RP intensity quantification in vasculature of EC-*Foxc2*-KO mice (*n* = 5) compared to littermate controls (*n* = 4). Data are mean ± SD. ns, not significant. Two-tailed unpaired *t* test. **G** Representative images of IB4 and pS6-RP staining of retina vasculature in P6 littermate control and EC-*Foxc1; Foxc2*-DKO individuals. Mean pS6-RP intensity quantification in vasculature of EC-*Foxc1; Foxc2*-DKO mice (*n* = 7) compared to littermate controls (*n* = 6). Data are mean ± SD. **p = 0.0041. Two-tailed unpaired *t* test. **H** Representative images of IB4 and pS6-RP staining of retina vasculature in P6 littermate control and EC-*Foxc1*^*fl/+*^*; Slc3a2*^*fl/+*^*; Slc7a5*^*fl/+*^-KO individuals. Mean pS6-RP intensity quantification in vasculature of EC-*Foxc1*^*fl/+*^*; Slc3a2*^*fl/+*^*; Slc7a5*^*fl/+*^-KO individuals (*n* = 9) compared to littermate controls (*n* = 8). Data are mean ± SD. *p = 0.0456. Two-tailed, unpaired *t* test. Scale bars are 100 μm. Source data are provided as Source Data file.

To investigate transcriptomic changes associated with endothelial loss of *Foxc1*, we performed differential expression analysis of RNA-sequencing data from isolated retinal endothelial cells collected from pooled tissue samples of EC-*Foxc1-*KO mice and littermate controls. The purity of isolated endothelial cells from P6 retinas was determined by staining with CD31 and CD45 (Supplementary Fig. 5). 426 differentially expressed genes (DEGs) with an FDR-adjusted *p* value of < 0.05 were significantly downregulated whereas 33 DEGs were significantly upregulated in EC-*Foxc1*-KO mice compared to littermate controls (Supplementary Data 1 and Fig. 4A). To identify possible relevant biological pathways associated with impaired angiogenesis as a result of loss of endothelial *Foxc1*, we performed gene ontology (GO) analysis on DEGs with a log2 fold change of ≤ −1 as only 4 DEGs had a log2 fold change ≥ 1 (Supplementary Data 1). The top GO term associated with loss of EC-*Foxc1* was “SLC-mediated transmembrane transport” (Fig. 4B) and we observed that the solute carrier (SLC) family members *Slc3a2* and *Slc7a5* were included within the top downregulated DEGs with the largest log2 fold change as a result of loss of *Foxc1* within the retina endothelium (Fig. 4A). *Slc3a2* and *Slc7a5* encode the 4F2hc (CD98 heavy subunit) protein and LAT1 (CD98 light subunit) protein, respectively, which assemble into the heterodimeric CD98 transporter^21,22^. In partial validation of our RNA-seq analysis, we also observed reduced protein expression in whole-retina lysates of the FOXC1 and LAT1 (Fig. 4C).

CD98 is a bidirectional amino acid transporter, exchanging intracellular glutamine for essential amino acids, which regulates activation of the mTORC1 signaling complex to promote cell growth^23,24^. Expression analysis of *Slc3a2* and *Slc7a5* in the single-cell portal (https://singlecell.broadinstitute.org/single_cell) showed that in addition to retinal ECs, both *Slc3a2* and *Slc7a5* are expressed in various cell types of the murine retina^25–27^. Notably, Gene Set Enrichment Analysis (GSEA) revealed a significant reduction in the gene set of mTORC1 signaling, including S6K, in the retinal ECs of EC-*Foxc1*-KO mice compared to the control mice (Fig. 4D). To investigate whether mTOR signaling is affected by the loss of *Foxc1*, we assessed the expression of phosphorylated S6 ribosomal protein (pS6-RP), which is a downstream target of mTOR effector ribosomal protein S6 kinase (S6K) and has previously been shown to have strong expression in the angiogenic retinal endothelium^28,29^. EC-specific deletion of *Foxc1* resulted in a significant reduction of pS6-RP in the retina vasculature at P6 (Fig. 4E), while the levels of pS6 in the retinal ECs of EC-*Foxc2*-KO mice were comparable to the controls (Fig. 4F). We also found that both EC-*Foxc1;Foxc2*-DKO and compound EC-*Foxc1;Slc3a2;Slc7a5* heterozygous mutant mice also had reduced S6 levels (Fig. 4G, H). These results indicate that FOXC1 is a key upstream regulator of mTOR and cellular growth in the retinal vasculature.

**Fig. 5:**
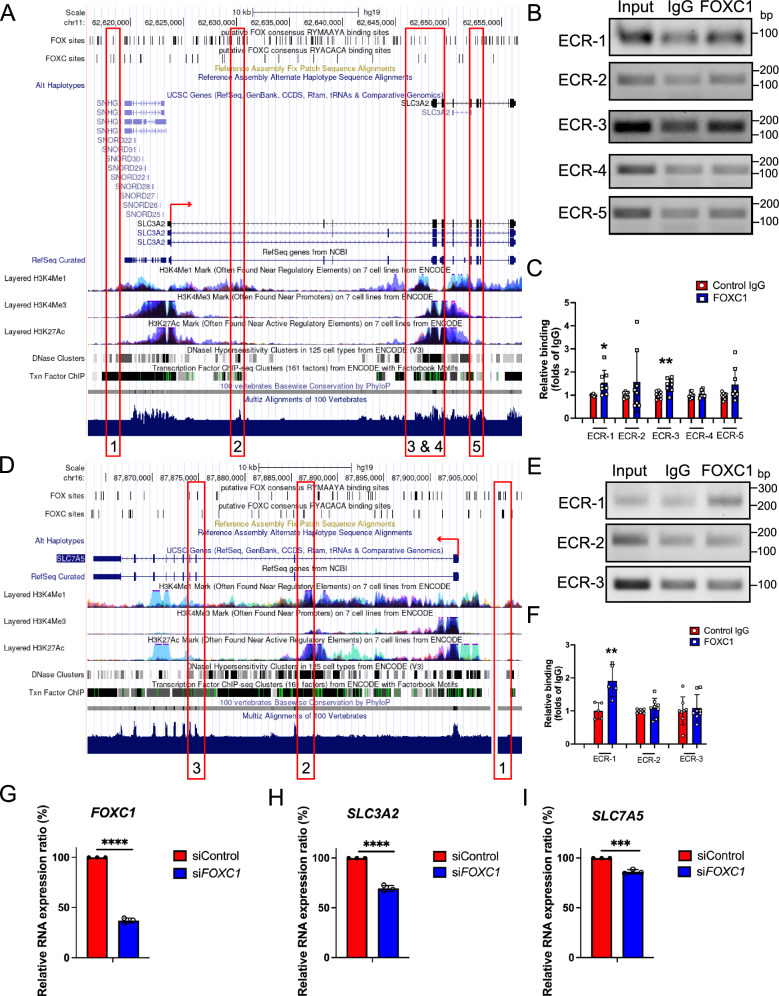
FOXC1 mediates retina endothelial expression of the bidirectional amino acid transporter 4F2hc/LAT1. **A**, **D** Putative FOX-binding sites in regions of the human *SLC3A2* or *SLC7A5* locus as viewed on the UCSC genome browser (http://genome.ucsc.edu). Vertical lines on the ‘FOX sites’ and ‘FOXC sites’ track indicate putative FOX-binding sites predicted using HOMER (see Materials and Methods). Red boxes indicate evolutionarily conserved regions (ECRs) containing FOX-binding sites between human and mouse genomes that are conserved and aligned. **B**, **E** ChIP showing specific binding of FOXC1 to the consensus FOX-binding sites within ECRs 1, 2, 3, 4, and 5 in *SLC3A2* or ECRs 1, 2, and 3 in *SLC7A5* in HRMVECs. **C**, **F** ChIP assays were performed using antibodies against FOXC1 and normal goat IgG. The binding of FOXC1 to candidate ECRs was determined with regular PCR and expressed as relative folds of input whose band intensity was normalized to 1. *n* = 8 biological duplicates from 4 independent experiments. Data are presented as mean ± SD. *p = 0.0142, **p = 0.0026. Student’s two-tailed, unpaired *t* test (**C**). *n* = 5 from 3 independent experiments for ECR-1 and *n* = 8 from 4 independent experiments for ECR-2 and ECR-3. Data are presented as mean ± SD. **p = 0.0057. Two-tailed, unpaired *t* test (**F**). RT-qPCR analysis of the siRNA-mediated knockdown of *FOXC1* in HUVECs resulting in reduced mRNA expression levels of (**G**) *FOXC1* (**H**) *SLC3A2* and (**I**) *SLC7A5*. *n* = 3 independent experiments. Data are presented as mean ± SD. ***p = 0.0004, ****p < 0.0001. Two-tailed unpaired *t* test is used for statistical analysis. Source data are provided as a Source Data file.

To confirm whether FOXC1 is capable of directly regulating *SLC3A2* and *SLC7A5* transcription, we first performed in silico analysis to identify putative FOXC-binding sites in the human *SLC3A2* and *SLC7A5* loci containing the FOX RYMAAYA consensus DNA binding sequence or FOXC RYACACA sequence as described previously in our group’s work identifying FOX-binding sites in the *PRICKLE1, ARHGAP21*, and *ARHGAP23* loci in human dermal lymphatic endothelial cells^18,30–32^. Several putative FOX-binding sites were identified in transcriptionally active, evolutionary conserved regions (ECRs) of the *SLC3A2* and *SLC7A5* loci whose sequences are conserved and aligned between the mouse and human genomes (Fig. 5A, D). ChIP assays performed in human retina microvascular endothelial cells (HRMVECs) using an antibody for FOXC1 showed that FOXC1 binding was significantly enriched in ECR-1 and -3 of *SLC3A2* (Fig. 5B, C) as well as ECR-1 of *SLC7A5* (Fig. 5E, F). Moreover, siRNA-mediated knockdown of *FOXC1* in human umbilical vein endothelial cells (HUVECs) resulted in reduced expression of both *SLC3A2* and *SLC7A5* (Fig. 5G–I).

**Fig. 6:**
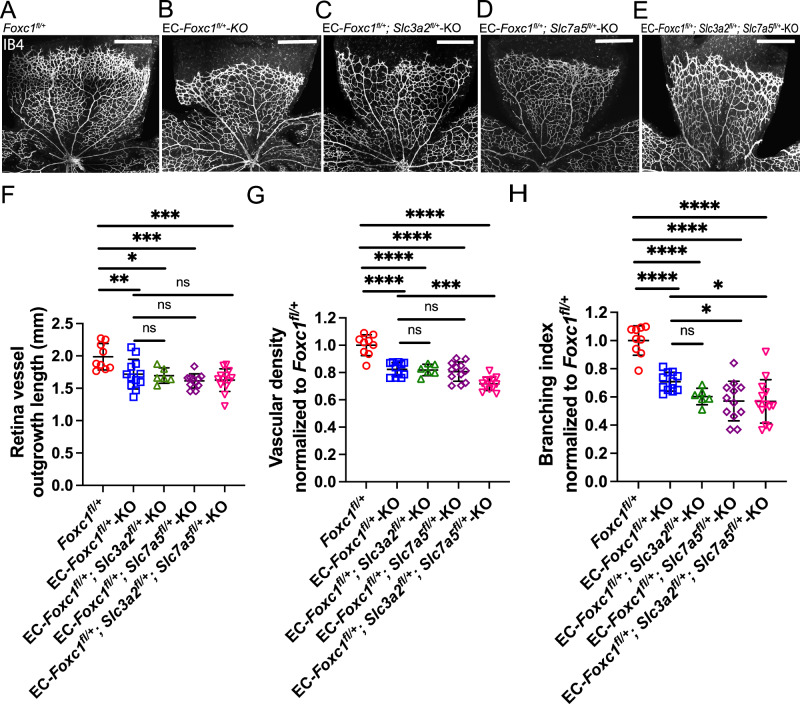
Compound endothelial-specific haploinsufficiency of *Foxc1* and *Slc3a2*, *Slc7a5*, or *Slc3a2* and *Slc7a5* impairs developmental retina angiogenesis. Representative images of IB4 staining in the retina vasculature of P6 *Foxc1*^*fl/+*^ (**A**), EC- *Foxc1*^*fl/+*^-KO (**B**) EC- *Foxc1*^*fl/+*^*; Slc3a2*^*fl/+*^-KO (**C**) EC- *Foxc1*^*fl/+*^*; Slc7a5*^*fl/+*^-KO (**D**) EC-*Foxc1*^*fl/+*^*; Slc3a2*^*fl/+*^*; Slc7a5*^*fl/+*^-KO (**E**). Scale bars are 500 μm. **F** Quantification of retina vascular outgrowth in P6 *Foxc1*^*fl/+*^(*n* = 9), EC- *Foxc1*^*fl/+*^-KO (*n* = 12), EC- *Foxc1*^*fl/+*^*; Slc3a2*^*fl/+*^ (*n* = 6), EC- *Foxc1*^*fl/+*^*; Slc7a5*^*fl/+*^-KO (*n* = 11) and EC-*Foxc1*^*fl/+*^*; Slc3a2*^*fl/+*^*; Slc7a5*^*fl/+*^-KO (*n* = 13) mice. Data are presented as mean ± SD. ns, not significant; *p = 0.0119, **p = 0.0049, ***p = 0.0001. One-way ANOVA with Dunnett’s multiple comparisons test. **G** Quantification of vascular density per 10X HPF in EC- *Foxc1*^*fl/+*^-KO (*n* = 11), EC- *Foxc1*^*fl/+*^*; Slc3a2*^*fl/+*^(*n* = 6), EC- *Foxc1*^*fl/+*^*; Slc7a5*^*fl/+*^-KO (*n* = 12) and EC-*Foxc1*^*fl/+*^*; Slc3a2*^*fl/+*^*; Slc7a5*^*fl/+*^-KO (*n* = 12) mice compared to *Foxc1*^*fl/+*^ control group (*n* = 9). Data are presented as mean ± SD. ns, not significant; ***p = 0.0005, ****p < 0.0001. One-way ANOVA with Dunnett’s multiple comparisons test. **H** Quantification of branching index per 10X HPF in EC- *Foxc1*^*fl/+*^-KO (*n* = 11), EC- *Foxc1*^*fl/+*^*; Slc3a2*^*fl/+*^ (*n* = 6), EC- *Foxc1*^*fl/+*^*; Slc7a5*^*fl/+*^-KO (*n* = 12) and EC-*Foxc1*^*fl/+*^*; Slc3a2*^*fl/+*^*; Slc7a5*^*fl/+*^-KO (*n* = 13) mice compared to *Foxc1*^*fl/+*^ control group (*n* = 9). Data are mean ± SD. ns, not significant; *p < 0.05, ****p < 0.0001. One-way ANOVA with Dunnett’s multiple comparisons test. Source data are provided as a Source Data file.

To further explore the genetic interaction of *Foxc1* with *Slc3a2* and *Slc7a5*, we generated inducible, endothelial-specific, compound heterozygous mutant mice for *Foxc1* and *Slc3a2* (EC-*Foxc1*^*fl/+*^*; Slc3a2*^*fl/+*^-KO), *Foxc1* and *Slc7a5* (EC-*Foxc1*^*fl/+*^*; Slc7a5*^*fl/+*^-KO) or all three genes (EC-*Foxc1*^*fl/+*^*; Slc3a2*^*fl/+*^*; Slc7a5*^*fl/+*^-KO) (Fig. 6A–E). Following tamoxifen administration from P1–P5, analysis of the retinal vasculature at P6 showed that EC-*Foxc1*^*fl/+*^-KO mice exhibited a reduction in retinal vessel outgrowth, as well as vascular density and branching in the capillary plexus compared to the control (*Foxc1*^*fl/+*^) mice (Fig. 6F–H), indicating that loss of one allele of *Foxc1* in ECs is sufficient to lower retinal vessel growth. Furthermore, retinal vessel outgrowth, vascular density, and branching were significantly reduced in all three EC-specific compound mutant (EC-*Foxc1*^*fl/+*^*; Slc3a2*^*fl/+*^-KO, EC-*Foxc1*^*fl/+*^*; Slc7a5*^*fl/+*^-KO, and EC-*Foxc1*^*fl/+*^*; Slc3a2*^*fl/+*^*; Slc7a5*^*fl/+*^-KO) backgrounds. While EC-*Foxc1*^*fl/+*^*; Slc3a2*^*fl/+*^*; Slc7a5*^*fl/+*^-KO mice showed a significant decrease in vascular density and branching compared to the EC-*Foxc1*^*fl/+*^-KO mice, branching was only reduced in EC-*Foxc1*^*fl/+*^*; Slc7a5*^*fl/+*^-KO mice (Fig. 6F–H). These phenotypic differences among the compound mutants may be partly because *Slc7a5* is expressed higher than *Slc3a2* in retinal ECs (Supplementary Data 1) and SLC3A2 acts as the ancillary subunit of the heterodimeric CD98 transporter^21,22^.

Collectively, these data demonstrate that *Slc3a2* and *Slc7a5* are key transcriptional targets of FOXC1 in the retinal vasculature that mediate angiogenic growth and development. Moreover, impaired retinal angiogenesis as a result of loss of *Foxc1* is in part associated with reduced mTOR signaling activity and cell growth.

### Stimulation of mTOR signaling partially rescues impaired angiogenesis in EC-*Foxc1*-KO mice

**Fig. 7:**
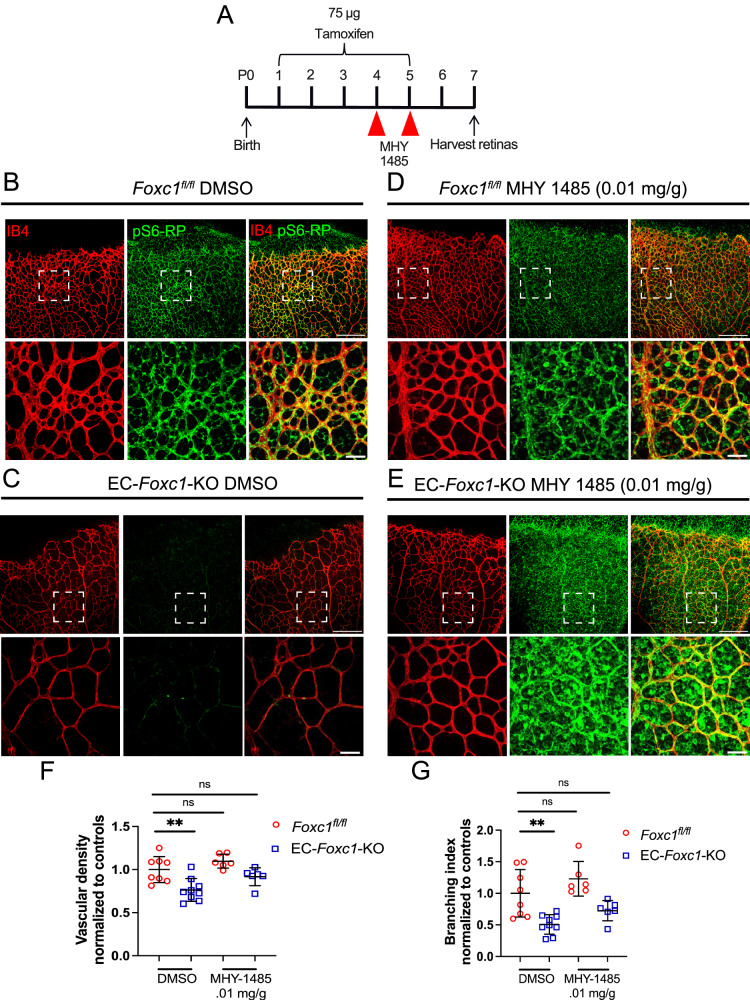
Stimulation of mTOR signaling partially rescues impaired angiogenesis in EC-*Foxc1*-KO mice. **A** Schematic diagram representing the time course of tamoxifen and MHY-1485 administration during experimental analysis. Representative images of IB4 and pS6-RP staining in the retina vasculature of P7 control and EC-*Foxc1*-KO mice treated with DMSO (**B**, **C**) or MHY-1485 (**D**, **E**). White boxes denote magnified regions depicted in lower panels. Scale bars are 200 and 50 µm respectively. **F** Quantification of vascular density per 10X HPF in the retina vasculature of P7 EC-*Foxc1*-KO mice treated with vehicle (*n* = 9) or MHY-1485 (*n* = 6) compared to littermate controls treated with vehicle (*n* = 8) or MHY-1485 (*n* = 6). Data are mean ± SD. ns, not significant; **p = 0.0017. One-way ANOVA with Dunnett’s multiple comparisons test. **G** Quantification of branching index per 10X HPF in the retina vasculature of P7 EC-*Foxc1*-KO mice treated with vehicle (*n* = 9) or MHY-1485 (*n* = 6) compared to littermate controls treated with vehicle (*n* = 8) or MHY-1485 (*n* = 6). Data are mean ± SD. ns, not significant; **p = 0.0018. One-way ANOVA with Dunnett’s multiple comparisons test. Source data are provided as a Source Data file.

Because pS6-RP expression was reduced in EC-*Foxc1-*KO mice (Fig. 4E), we hypothesized that stimulation of mTOR signaling may rescue the impaired endothelial proliferation and angiogenesis phenotype. To investigate this, we administered the chemical compound MHY-1485, which has previously been shown to stimulate mTOR^33^, to neonatal mice at P4 and P5, concurrent with tamoxifen administration from P1–P5, and assessed the retina vasculature at P7 (Fig. 7A). As compared to Cre-negative controls administered with DMSO (Fig. 7B), EC-*Foxc1*-KO mice administered with DMSO vehicle exhibited reduced pS6-RP expression (Fig. 7C) and exhibited significantly reduced capillary plexus density and branching (Fig. 7F, G). However, administration of MHY-1485 to EC-*Foxc1*-KO mice robustly increased pS6-RP expression in the retinal vasculature (Fig. 7E) and no statistically significant differences related to capillary density or branching were observed in these individuals compared to Cre-negative controls administered DMSO vehicle (Fig. 7F, G). In contrast, administration of MHY-1485 to Cre-negative controls resulted in a trend towards increased vascular density and branching, although it was not statistically significant (Fig. 7F, G). Thus, regulation of mTOR signaling activity and cell growth is a critical function of FOXC1 transcriptional activity in the retinal vasculature during sprouting angiogenesis.

### FOXC1 is required for physiological revascularization in a model of oxygen-induced retinopathy

**Fig. 8:**
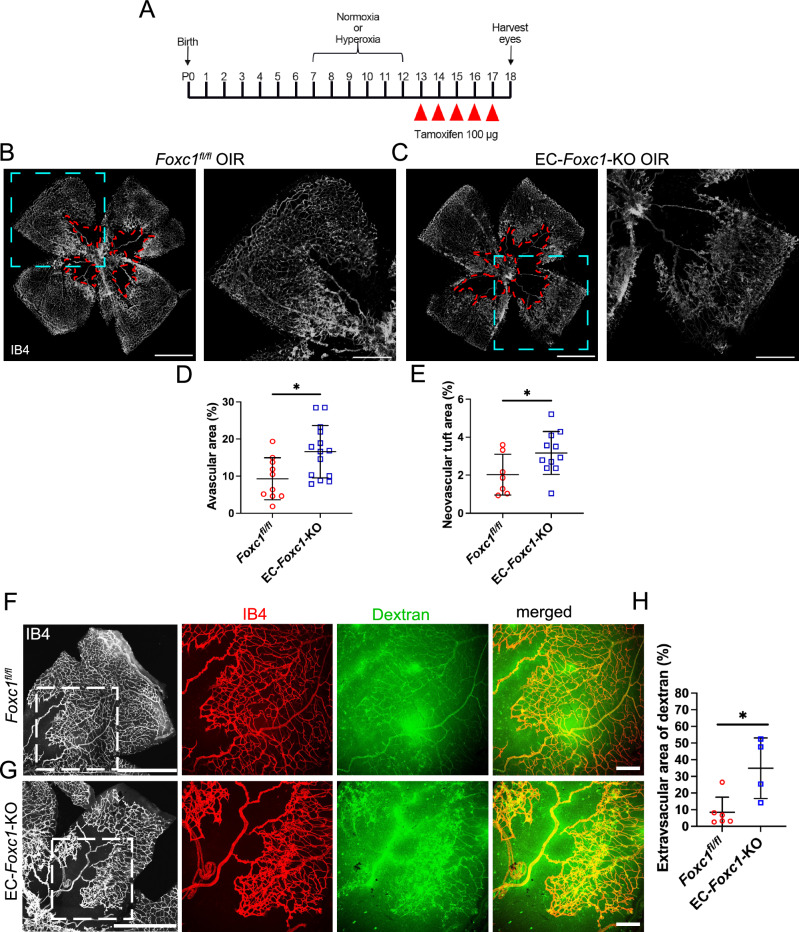
Endothelial FOXC1 is required for physiological revascularization of the retina during OIR. **A** Schematic of experimental analysis to characterize the role of endothelial FOXC1 during late stages of developmental retina angiogenesis and pathological neovascularization during OIR. Representative images of IB4 stained retina vasculature in P18 littermate control (**B**) and EC-*Foxc1*-KO (**C**) mice exposed to hyperoxia conditions. Dashed red lines highlight avascular regions. Boxed regions denote magnified images in the right panels. Scale bars are 1 mm and 500 μm respectively. **D** Quantification of % avascular area in P18 EC-*Foxc1*-KO mice (*n* = 14) compared to littermate controls (*n* = 10). Data are mean ± SD. *p = 0.0129. Two-tailed unpaired *t* test. **E** Quantification of % neovascular tuft area in P18 EC-*Foxc1*-KO mice (*n* = 11) compared to littermate controls (*n* = 7). Data are mean ± SD. *p = 0.0494. Two-tailed, unpaired *t* test. Representative images of retina vessel leakage assessed by retro orbital injections of FITC-dextran and IB4-568 in P18 littermate control (**F**) and EC-*Foxc1*-KO (**G**) mice exposed to OIR. Boxed regions denote magnified images in the right panels. Scale bars are 1 mm and 200 μm respectively. **H** Quantification of extravascular dextran leakage in the retinas of EC-*Foxc1*-KO (*n* = 4) and littermate controls (*n* = 6) mice exposed to OIR at P18. The dextran positive area subtracted from the IB4 positive area is regarded as the extravascular dextran leakage area and has been presented as %. Data are mean ± SD. *p = 0.0148. Two-tailed unpaired *t* test. Source data are provided as a Source Data file.

Our previous studies primarily focused on the contributions of endothelial-derived FOXC1 transcriptional regulation during early postnatal retina angiogenesis. However, the role of endothelial-derived FOXC1 transcriptional activity both during late stages of retina vascular development and during pathological angiogenesis and neovascularization is not well understood. To address this, EC-*Foxc1*-KO mice and littermate controls were subjected to either normoxia conditions from P7 – P12, or kept under hyperoxia (75% oxygen) to induce retinal vessel regression and vaso-obliteration. Subsequently, these pups were returned to room air conditions from P13 – P17, which is associated with pathological neovascularization in phase 2 of OIR^15,34,35^. To investigate the role of *Foxc1* in the late stages of developmental retina angiogenesis, or during OIR, tamoxifen was administered to EC-*Foxc1*-KO mice and littermate controls from P13 – P17, following normoxia or OIR protocols (P7 – P12), and the retinal vasculature was assessed at P18 (Fig. 8A). Characterization of the superficial and deep retinal vascular plexuses in littermate control and EC-*Foxc1*-KO mice kept under normoxia conditions showed no obvious morphological differences (Supplementary Fig. 6A–D) and quantification of vascular density in retinal flatmounts showed no significant differences (Supplementary Fig. 6E). In P18 mice, FOXC1 was strongly expressed within arterioles, whereas venule and capillary endothelium showed modest FOXC1 nuclear expression. Additionally, FOXC1 nuclear expression was observed in vascular-associated mural cells (Supplementary Fig. 7A). In validation of Cre-recombination efficiency and specificity at P18 with tamoxifen administration from P13 – P17, FOXC1 expression was strongly reduced in the retinal vascular endothelium, but FOXC1-positive expression was observed in mural cells (Supplementary Fig. 7B). Thus, while FOXC1 transcriptional activity is critical during early postnatal retina angiogenesis (P1–P5), its activity is dispensable during later stages of retinal vascular maturation and maintenance (P13 – P17).

In contrast to mice kept under normoxic conditions, OIR mice exhibited vaso-obliteration in the central retinal region and neovascular tuft formation (Fig. 8B, C). Investigation of the retinal vasculature at P18 (following peak neovascularization at P17^15^) revealed that the avascular area was significantly greater in EC-*Foxc1*-KO mice (Fig. 8D) and the percentage of the retinal vasculature comprising neovascular tufts was significantly greater as well (Fig. 8E). Similar to the vascular leakage observed in the P6 retinal vessels of EC-*Foxc1*-KO mice during postnatal retinal angiogenesis (Fig. 3M–O), retinal vessels at P18 in the EC-*Foxc1*-KO OIR model were leaky compared to the control OIR vessels (Fig. 8F–H). Thus, EC-*Foxc1* is required for physiologic revascularization of the vaso-obliterated regions of the retina during phase 2 of OIR. Additionally, persistent pathological angiogenesis and increased neovascular tuft formation in EC-*Foxc1*-KO mice may be secondary to the delay in physiologic revascularization of the retina. As a greater region of the retina in EC-*Foxc1*-KO mice remains ischemic, this may, in turn, stimulate greater production of VEGF and other pro-angiogenic factors, which can stimulate vascular leakage, vasoproliferation and neovascular tuft formation^15,36^.

### FOXC2 is capable of functionally substituting for FOXC1 during retina angiogenesis

**Fig. 9:**
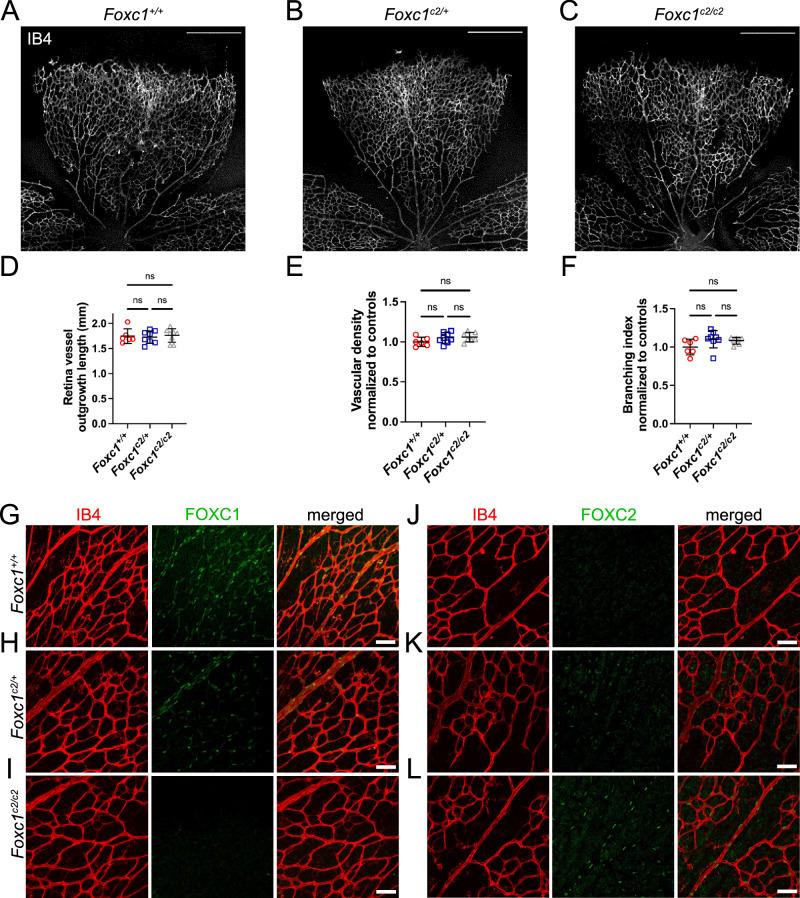
FOXC2 is able to functionally substitute for FOXC1 during developmental retina angiogenesis. Representative images of IB4 stained retina vasculature in P6 *Foxc1*^*+/+*^ (**A**), *Foxc1*^*c2/+*^ (**B**), and *Foxc1*^*c2/c2*^ (**C**) mice. Scale bars are 500 µm. **D** Quantification of retina vessel outgrowth length in P6 *Foxc1*^*+/+*^ (*n* = 6), *Foxc1*^*c2/+*^ (*n* = 8), and *Foxc1*^*c2/c2*^ (*n* = 8) mice. Quantification of vascular density per 10X HPF (**E**) and branching index per 10X HPF (**F**) in the rear capillary plexus of P6 *Foxc1*^*+/+*^ (*n* = 6), *Foxc1*^*c2/+*^ (*n* = 8), and *Foxc1*^*c2/c2*^ (*n* = 7) mice. Data are mean ± SD. ns, not significant. One-way ANOVA, Tukey’s multiple comparisons test. Representative images of IB4 and FOXC1 stained retina vasculature in P6 *Foxc1*^*+/+*^ (**G**), *Foxc1*^*c2/+*^ (**H**), and *Foxc1*^*c2/c2*^ (**I**) mice. Scale bars are 50 µm. Representative images of IB4 and FOXC2 stained retina vasculature in P6 *Foxc1*^*+/+*^ (**J**), *Foxc1*^*c2/+*^ (**K**), and *Foxc1*^*c2/c2*^ (**L**) mice. Scale bars are 50 µm. Source data are provided as a Source Data file.

FOXC1 and FOXC2 share nearly identical forkhead DNA binding domains and our laboratory has demonstrated both cooperative and complementary roles for these transcription factors during early cardiovascular^9^ and ocular^37^ development, embryonic lymphangiogenesis^38^, and postnatal lymphatic collecting vessel valve maturation and maintenance^18^, as well as during intestinal regeneration^39^. We previously reported that transcriptional function in the development of lymphatic collecting vessels was conserved in a genetic knock-in model where the *Foxc1* coding region was faithfully replaced with *Foxc2* cDNA (*Foxc1*^*c2*^ mice), but endothelial-specific deletion of *Foxc1* resulted in a modest, impaired valve maturation phenotype^18^. To investigate whether FOXC2 transcriptional activity could similarly functionally substitute for FOXC1, we generated wildtype (*Foxc1*^*+/+*^), heterozygous (*Foxc1*^*c2/+*^), and homozygous (*Foxc1*^*c2/c2*^) knock-in mice to assess retinal angiogenesis at P6 (Fig. 9). No obvious morphological differences were observed in the retinal vasculature of these individuals (Fig. 9A–C) and quantification of vessel outgrowth, vascular density, and branching did not identify significant differences (Fig. 9D–F). Immunostaining analysis of FOXC1 and FOXC2 expression validated loss of FOXC1 nuclear expression in the vascular endothelium and mural cells with loss of *Foxc1* alleles (Fig. 9G–I) and subsequent gain of FOXC2 nuclear expression (Fig. 9J–L). Thus, like our previously reported results^18^, FOXC2 is capable of functioning for FOXC1 in the regulation of vascular development and this functional ability is conserved among different vascular beds.

### Pericyte-specific loss of FOXC1 impairs the blood-brain barrier in postnatal retina angiogenesis

**Fig. 10:**
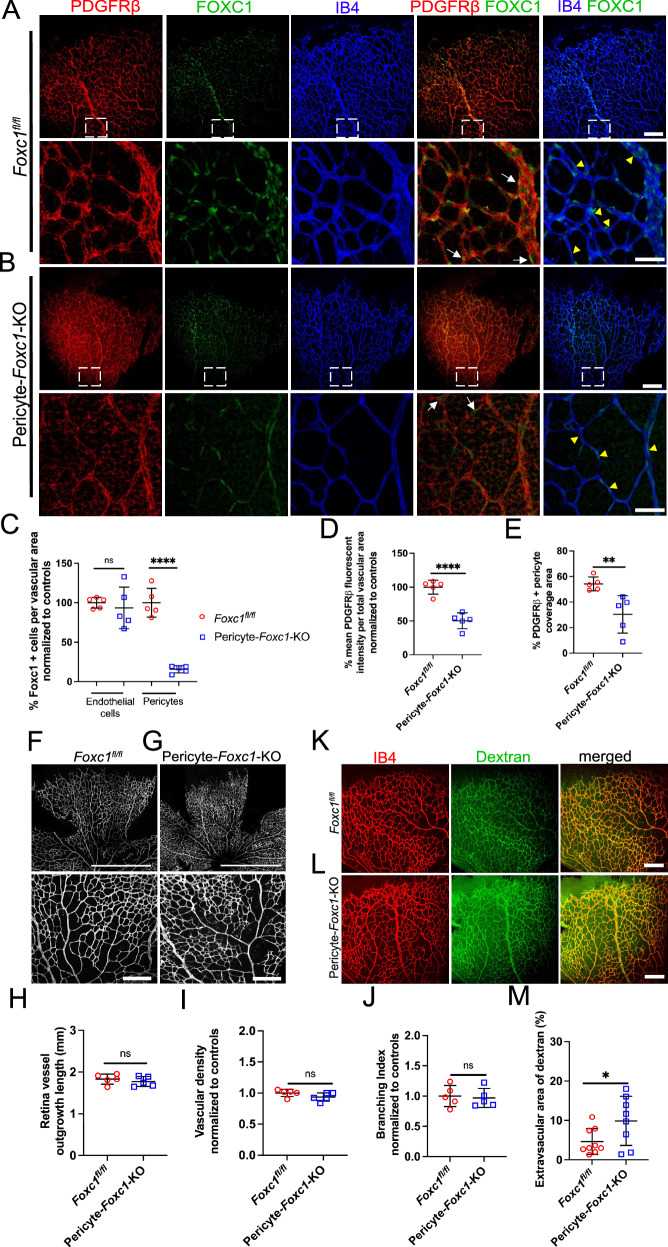
Loss of pericyte-specific expression of *Foxc1* impairs the blood-retina barrier in postnatal retina angiogenesis. Representative images of PDGFRβ, FOXC1, and IB4 staining in the retina vasculature of P6 control (**A**) and Pericyte-*Foxc1*-KO (**B**) individuals. White boxes denote magnified regions depicted in lower panels. White arrows denote FOXC1+ pericytes and yellow arrowheads denote FOXC1+ endothelial cells in both control and Pericyte-*Foxc1*-KO mutant mice. Scale bars are 200 μm and 50 μm respectively. **C**, **D**, **E** Quantification of FOXC1-positive cells in the endothelial cells and pericytes per vascular area in P6 Pericyte-*Foxc1*-KO (*n* = 5) and littermate controls (*n* = 5). Data are mean ± SD. ns, not significant; ****p < 0.0001. Two-tailed unpaired *t* test (**C**). % mean PDGFRβ fluorescent intensity per total vascular area in P6 Pericyte-*Foxc1*-KO (*n* = 5) and littermate controls (*n* = 5). Data are mean ± SD. ****p < 0.0001. Two-tailed unpaired *t* test (**D**). % PDGFRβ+ pericyte coverage area in P6 Pericyte-*Foxc1*-KO (*n* = 5) and littermate controls (*n* = 5). Data are mean ± SD. **p = 0.0090. Two-tailed unpaired *t* test (**E**). Representative images of isolectin-B4 (IB4) staining of retina leaflets and the rear capillary plexus of P6 littermate control (**F**) and Pericyte-*Foxc1*-KO (**G**) mice. Scale bars are 1 mm and 200 μm respectively. **H** Quantification of retina vessel outgrowth length, vascular density per 10X HPF (**I**), and branching index per 10X (**J**) in P6 Pericyte-*Foxc1*-KO (*n* = 5) mice compared to littermate controls (*n* = 5). Data are mean ± SD. ns, not significant. Two-tailed unpaired *t* test. **K**, **L** Representative images of retina vessel leakage assessed by retro orbital injections of FITC-dextran and IB4-568 in P6 littermate control (**K**) and Pericyte-*Foxc1*-KO (**L**) individuals. Scale bars are 200 μm. **M** Quantification of extravascular dextran leakage in the retinas of Pericyte-*Foxc1*-KO (*n* = 8) and littermate control (*n* = 9) mice at P6. The dextran positive area subtracted from the IB4 positive area is regarded as the extravascular dextran leakage area and has been presented as %. Data are mean ± SD. *p = 0.0427. Two-tailed unpaired *t* test. Source data are provided as a Source Data file.

As described above, FOXC1 is also expressed in retinal pericytes, a major component of the blood-retina barrier (BRB). To determine the pericyte-specific roles of *Foxc1* in retinal vessels, we generated and analyzed pericyte-specific *Foxc1*-mutant (*PDGFRβ-P2A-CreERT2; Foxc1*^*fl/fl*^) mice (Fig. 10A–C). These mutant mice exhibited a significant reduction in the levels of PDGFRβ expression and pericyte coverage in the retina (Fig. 10D, E), whereas retinal capillary density and overall vascular indices were comparable to the control mice (Fig. 10F–J). Most importantly, vascular leakage at P6 was significantly increased in the pericyte-specific *Foxc1* mutants (Fig. 10K–M). These results suggest that FOXC1 plays a pivotal role in the establishment of pericyte coverage and the maintenance of the BRB.

## Discussion

FOXC1/2 transcription factors are key regulators of developmental and functional processes, including growth and maintenance of the blood and lymphatic vasculature as the endothelial-specific roles^18,20,38–40^. While more attention has been focused on the role of FOXC1/2 in lymphatic vascular development given the clinical relevance of *FOXC2* mutations in lymphedema distichiasis^12^, the role of FOXC-dependent transcriptional regulation in blood vascular development and angiogenesis is not well understood. Here, we demonstrate that retinal angiogenesis is primarily regulated by FOXC1 transcriptional activity. EC-specific deletion of *Foxc1* impaired retinal angiogenesis in mice whereas *Foxc2* deletion only resulted in comparatively minor phenotypic differences. Moreover, our evidence shows that compound deletion of *Foxc1/2* does not result in an additive phenotype compared to a single deletion of *Foxc1*, yet these mice more rapidly develop severe defects in lymphatic collecting vessels compared to a single deletion of *Foxc2*^18^. Importantly, we also show that endothelial FOXC1 is a key regulator in the retinal vasculature via directly controlling expression of *Slc3a2* and *Slc7a5*, which encode the heterodimeric 4F2hc/LAT1 (CD98) amino acid transporter (Supplementary Fig. 8). While early deletion of FOXC1 impaired angiogenesis from nascent vessels, deletion during later stages of retinal angiogenesis revealed that FOXC1 is dispensable for maturation and maintenance of established retinal vasculature. However, in the OIR model, EC-*Foxc1* deletion impaired physiological revascularization of the retina and disrupted the maintenance of the BRB, potentially contributing to impaired resolution of neovascular tuft formation. Moreover, *Foxc1* expression in retinal pericytes is required for proper pericyte coverage and BRB formation during postnatal retinal angiogenesis. Collectively, our findings reveal that FOXC1 is a key transcriptional regulator for blood vessel growth as well as the BRB in the retinal vasculature.

Previous studies demonstrated a role for FOXC1 function in vascular development. Poor integrity of blood vessels and blood-stained hydrocephalus were observed in murine *Foxc1*^*-/-*^ embryos^8^, zebrafish treated with *foxc1* morpholino exhibited several developmental vascular defects^41^ as well as cerebral hemorrhage^11,41^, and several *FOXC1*-attributable ARS patients exhibited CSVD phenotypes^11^. In zebrafish models of *foxc1* morpholino treatment, defects in the retinal vasculature were attributable to disrupted basement membrane structure^41^. Our RNA-seq analysis of isolated retinal endothelial cells shows significant reduction of *Lama4* (encoding Laminin, alpha 4) and *Plod1* (encoding lysyl hydroxylase 1) (Supplementary Data 1), suggesting that basement membrane structural components may be affected in EC-*Foxc1*-KO mice. However, the cerebrovascular defects observed in zebrafish treated with *foxc1* morpholino may also be attributable to perturbed perivascular function as *foxc1* morpholino treatment reduced cerebral vascular smooth muscle cell numbers^11^ and conditional deletion of *Foxc1* from pericytes and vascular smooth muscle cells led to late-gestation cerebral micro-hemorrhages and pericyte and endothelial cell hyperplasia as a result of alterations in pericyte-expressed proteoglycans in mice^42^. It was previously suggested that neural-crest-derived meninges require *Foxc1* for proper development and production of retinoic acid to regulate WNT pathway proteins and neural progenitor-derived VEGF-A to promote cerebrovascular development^43^. Surprisingly, this same study also suggested that endothelial-derived *Foxc1* is not required for cerebrovascular development whereas we demonstrate a key *Foxc1* requirement in the retinal vasculature, which shares developmental origins with the brain vasculature^44,45^. A possible explanation for this discrepancy is that this previous study used heterozygous *Tie2-Cre; Foxc1*^*fl/+*^ mice as controls to compare to homozygous *Tie2-Cre; Foxc1*^*fl/fl*^ knockout mice, and there is a lack of quantitative analysis comparing phenotypes between the two groups^43^. However, our data show that *Foxc1* is expressed in retinal ECs (Fig. 2, Supplementary Fig. 7, and Supplementary Data 1) and EC-*Foxc1* heterozygosity may be sufficient to impair retinal vascular growth and development (Fig. 6). It should also be noted that the pericyte-specific Foxc1-mutant mice used in this study do not display endothelial hyperplasia in the retinal vessels (Fig. 10F, G), contrary to the brain vasculature^42^. It is therefore possible that FOXC1 has a different role in the retinal vasculature compared to the brain vasculature. Nonetheless, a comparison of the role of FOXC1-dependent transcriptional regulation in these two different vascular beds warrants further investigation. Moreover, given the reduced expression of PDGFRβ and disrupted BRB in EC-Foxc1-mutant retinal vessels at P6 (Figs. 2D and 3M–O), determining how FOXC1’s transcriptional program in the endothelium and perivascular cells coordinate with one another to promote the growth and maturation of a functional vascular system will also be key for future studies.

The role of endothelial metabolism during physiological and pathological angiogenesis has recently gained more appreciation^4^. ECs are highly dependent on glutamine metabolism to generate critical intermediates for the tricarboxylic acid (TCA) cycle and inhibition of glutaminase 1 (GLS1), the enzyme converting glutamine to glutamate, impairs EC proliferation^46,47^. Glutamine depletion in ECs subsequently impairs both protein and nucleotide synthesis and mTOR activity^46^. Notably, our RNA-seq analysis and ChIP studies identified the SLC family members, *Slc3a2* and *Slc7a5* as important targets of FOXC1 transcription in blood ECs (BECs). CD98/LAT1 is a critical regulator of L-glutamine flux and uptake of exogenous, essential amino acids to stimulate activation of the mTORC1 signaling complex localized to lysosomal membranes, thus promoting cellular growth (Supplementary Fig. 8)^23,24^. Conditional EC-specific *Slc3a2* knockout mice are characterized by impaired retinal angiogenesis, which was attributed to observations that the 4F2hc integrin signaling domain is key for activation of VEGFR-2 signaling^48^. Another study investigating the role of endothelial *Slc7a5* identified that conditional deletion resulted in reduced uptake of branched-chain amino acids (BCAA) in the brains of adult mice compared, suggesting that *Slc7a5* has a key role in blood-brain barrier function^49^. Here, Tarlungeanu et al. demonstrated that loss of endothelial *Slc7a5* was associated with motor delay and autism-related phenotypes in mice. Moreover, this study identified two families with several children affected by autism spectrum disorder harboring homozygous missense mutations in *Slc7a5*. Notably, clinical studies of ARS patients have identified a number of presenting features characteristic of intellectual disability or autism^50,51^. Thus, potential loss of *Slc3a2* and *Slc7a5* expression in the cerebral vasculature and loss of BCAA uptake in the brain may impair cognitive ability in patients with FOXC1-associated mutations.

In contrast to previous studies investigating the role of endothelial *Slc3a2* and *Slc7a5*, our results demonstrating reduced pS6-RP expression in the retinal vasculature of EC-*Foxc1-*KO mice as well as EC-*Foxc1*^*fl/+*^*; Slc3a2*^*fl/+*^*; Slc7a5*^*fl/+*^-KO mice, and subsequent rescue of impaired retinal vascular growth with an mTOR agonist shows that mTOR signaling is critically regulated by FOXC1’s transcriptional activity. Previous studies have characterized the key role of endothelial PI3K/AKT/mTOR activation in physiological and pathological angiogenesis^3^ and its regulation by upstream factors including VEGF, angiopoietin-1, insulin, and sphingosine-1-phosphate among others^52^. Thus, FOXC1’s function is likely to link with the crosstalk between the VEGF/VEGFR-2 and mTOR signaling pathways^53^. Recent evidence indicates that Yes-associated protein 1 (YAP) and WW domain-containing transcription regulator 1 (WWTR1, also known as TAZ), essential effectors of the Hippo signaling pathway, control the expression of SLC3A2 and SLC7A5 in ECs, thereby stimulating mTOR signaling in angiogenesis^54^. Since the initial impairments in retinal angiogenesis of EC-*Foxc1*-KO mice seem to recover at the later stages, it is plausible that other key pathways/factors that control mTOR signaling such as YAP/TAZ are involved in this recovery process as a compensatory mechanism. Activation of mTORC1 is also regulated by energy status not only related to amino acid metabolism but through sensing Glucose/Glycogen metabolism and the ratio of ATP to ADP and AMP^55^. In addition to the reduced expression of *Slc3a2* and *Slc7a5* in ECs (Supplementary Data 1, Fig. 5H, I), loss of endothelial FOXC1 is associated with reduced expression of several SLC family members, including *Slc2a1* and *Slc16a1* (Supplementary Data 1). *Slc2a1*, encoding the GLUT1 glucose transporter, is crucial for developmental central nervous system (CNS) angiogenesis and adult CNS homeostasis where inhibition of GLUT1 impaired mTOR signaling activity^56^. Notably, characterization of monocarboxylate transporter 1 (MCT1, encoded by *Slc16a1*) and GLUT1 expression in the retinal vasculature showed that MCT1 is predominately expressed in the neonatal retinal vasculature and there is a shift from MCT1 expression to GLUT1 after weaning. This likely coincides with the change in the source of nutrients in young mice as the developing retina may respond to the uptake of monocarboxylates derived from milk and those in the circulating blood, whereas GLUT1 may be induced in response to weaned mice acquiring nutrition from standard chow diets with greater carbohydrate content^57^. Thus, it is important to consider that FOXC1 may act as an important regulator of endothelial metabolism through transcriptional control of several critical nutrient transporters to affect mTORC1 activation and anabolic pathways.

Utilizing a transgenic model in which the *Foxc2* coding region was recombined into the *Foxc1* locus, we demonstrate that *Foxc2* is capable of functionally substituting for *Foxc1* in the physiological development of the retinal vasculature, similar to our group’s previous observations demonstrating that no phenotypic differences were detected in the mesenteric lymphatic collecting vessels^18^ and Schlemm’s canal endothelial cells^58^ of *Foxc1*^*c2/c2*^ mice. Since *Foxc1* transcripts are more highly expressed than *Foxc2* in neonatal retinal ECs (Supplementary Data 1), their expression levels appear important in retinal angiogenesis, and FOXC1 has a primary role in this process. However, how the expression of these two closely related FOXC transcription factors is regulated remains a key outstanding question. We previously reported that laminar shear stress forces induce both FOXC1 and FOXC2 expression in cultured lymphatic ECs (LECs) in vitro, whereas oscillatory shear stress more robustly induces FOXC2 expression^18^. Analysis of flow at single-cell resolution has demonstrated that the retinal vasculature is exposed to pulsatile laminar flow in mice, but that flow varies greatly depending on vessel diameter^59^. Our immunostaining analysis demonstrated that FOXC1 is expressed globally within the retinal vasculature, suggesting that FOXC1 and FOXC2 expression is not strongly regulated by mechanosensing mechanisms as was observed in lymphatic collecting vessels and valves. Thus, determining how the expression of *FOXC1* and *FOXC2* is regulated in different vascular systems is essential for future analyses.

In conclusion, the findings reported here demonstrate that FOXC1 is a key regulator of physiological retinal angiogenesis, through regulation of CD98 (LAT1/4F2hc) expression. Furthermore, FOXC1 regulates physiological revascularization and BRB formation in a model of retinopathy. Moreover, FOXC1 is required in retinal pericytes for proper pericyte coverage and BRB formation. These results not only advance the current understanding of transcriptional mechanisms contributing to physiological retina development but also identify amino acid metabolism as a potential therapeutic target for the treatment of FOXC1-associated vascular anomalies, such as CSVD.

## Methods

### Animal generation and husbandry

This research complies with the relevant ethical regulations. All animal procedures were approved by the Institutional Animal Care and Use Committee at Northwestern University.

Mice were housed and kept under normal lighting conditions with 12-h-on, 12-h-off cycles, 72 ± 2 °F temperature and 30−70% humidity range in the Center for Comparative Medicine at Northwestern University. *Slc3a2*^*fl/+*^, and *Slc7a5*^*fl/+*^were purchased from The Jackson Laboratory. EC-specific *Foxc1*, *Foxc2*, and compound *Foxc1, Foxc2* knockout mice and *Foxc1*^*c2/+*^ and *Foxc1*^*c2/c2*^ were generated as previously described^18^. *Slc3a2*^*fl/fl*^*, Slc7a5*^*fl/fl*^ and compound *Slc3a2*^*fl/fl*^*; Slc7a5*^*fl/fl*^ mice were generated by crossing *Slc3a2*^*fl/+*^ and *Slc7a5*^*fl/+*^ mice through several generations to acquire the desired genotypes. To generate compound heterozygous EC-*Foxc1*^*fl/+*^*; Slc3a2*^*fl/+*^-KO, EC-*Foxc1*^*fl/+*^*; Slc7a5*^*fl/+*^-KO, and EC-*Foxc1*^*fl/+*^*; Slc3a2*^*fl/+*^*; Slc7a5*^*fl/+*^-KO mice for analysis, *Cdh5-Cre*^*ERT2*^; *Foxc1*^*fl/fl*^ mice were crossed with *Slc3a2*^*fl/fl*^, *Slc7a5*^*fl/fl*^, or *Slc3a2*^*fl/fl*^*; Slc7a5*^*fl/fl*^ mice. *Foxc1*^*c2/+*^ mice were crossed with one another to generate *Foxc1*^*+/+*^, *Foxc1*^*c2/+*^, and *Foxc1*^*c2/c2*^ mice for analysis as previously described^18^. *Foxc1*^*+/+*^, *Foxc1*^*c2/+*^, and *Foxc1*^*c2/c2*^ mice were bred on a mixed (129 and Black Swiss mix) genetic background. PDGFRβ-P2A-CreERT2 mice^60^ purchased from the Jackson Laboratory (Stock #030201) were crossed with *Foxc1*^*fl/fl*^ mice to obtain pericyte-specific *Foxc1*-mutant (*PDGFRβ-P2A-CreERT2; Foxc1*^*fl/fl*^) mice. EC-specific *Foxc1*, *Foxc2*, compound *Foxc1, Foxc2* knockout mice, EC-*Foxc1*^*fl/+*^*; Slc3a2*^*fl/+*^-KO, EC-*Foxc1*^*fl/+*^*; Slc7a5*^*fl/+*^-KO, EC-*Foxc1*^*fl/+*^*; Slc3a2*^*fl+*^*; Slc7a5*^*fl/+*^-KO mice, and pericyte-specific *Foxc1* knockout mouse were bred on a mixed (C57BL/6 J and 129 mix) genetic background. Both male and female mice were used. To induce Cre-mediated recombination postnatally, tamoxifen dissolved in corn oil was orally administered (75 μg per neonate) daily from P1 to P5 as previously described^18^. For assessment during late stages of postnatal retina angiogenesis or OIR, 100 μg of tamoxifen diluted in corn oil was injected subcutaneously into pups daily from P13 to P17. In some experiments, mice received an intraperitoneal injection of 0.01 mg/g MHY-1485 (Sigma-Aldrich, SML0810) or DMSO diluted in corn oil once daily on P4 and P5 with analysis at P7. Tamoxifen-injected Cre-negative littermates were used as controls. Genotyping was performed by Transnetyx Inc. (Cordova, TN) using real-time PCR.

### Whole-mount immunohistochemistry

The retina blood vasculature was analyzed by whole mount immunostaining of retina tissue harvested from pups at the indicated time points as previously described^61^. Briefly, retina tissue was dissected from enucleated eyes in PBS. Following fixation with 4% PFA in PBS for 30 min. at room temperature, enucleated eyes were washed with PBS. Retina tissue was then dissected and permeabilized and blocked in blocking buffer consisting of 1% FBS, 3% BSA, 0.5% Triton X-100, 0.01% sodium deoxycholate, and 0.02% sodium azide in PBS pH = 7.4 for 1 h at room temperature on a rocking platform. Tissues were then incubated with primary antibodies against FOXC1 (Cell Signaling Technologies, 8758 S, 1:50), FOXC2 (kind gift from Dr. N Miura, Miura et al., 1997, *Genomics*, 1:200), phospho-Histone H3 (Abcam, ab5176, 1:100), Active caspase 3 (R&D, AF835, 1:100), phospho-S6 ribosomal protein (Ser235/236, Cell Signaling Technologies, 2211 S, 1:100/1:500) or CD140b (PDGFRB) (APB5, Thermo Fisher Scientific, 14-1402-81, 1:50) overnight followed by washing with PBS containing 0.2% Triton X-100 prior to incubation with Alexa Fluor 488- or Alexa Fluor 568-conjugated secondary antibodies (Thermo Fisher Scientific, A-21206; A-21208; A-11077; Abcam, ab175475, 1:500) and Isolectin-B4 (IB4) Alexa Fluor 488-, IB4 Alexa Fluor 568- or IB4 Alexa Fluor 647- conjugate (Thermo Fisher Scientific, I21411; I21412; I32450, 1:100) for 2 h. After subsequent washes, samples were flat mounted on slides in mounting media (Vectashield, Vector Laboratories). Mesentery tissue was dissected from neonatal individuals, fixed in 2% PFA, and processed for whole mount immunostaining as previously described^18^.

### BRB permeability assay

To assess retinal vascular leakage, pups were anesthetized using isoflurane and a mixture of 10 kDa-FITC-dextran, 100 mg/mL (Sigma, FD10S) and fluorescent Alexa568-conjugated IB4 (5 mg/kg) was injected retro-orbitally into EC-*Foxc1*-KO and littermate control P6 pups (2.8−4.1 g), EC-*Foxc1*-KO and littermate control P21 pups (5.9−12.05 g) and Pericyte-*Foxc1*-KO and littermate control P6 pups (2.4−3.9 g). The volume of the combined injections of dextran and IB4 was determined based on the body weight of each pup. Euthanization was performed after 10 min of circulation and eyes were enucleated. A small pinch was made in the cornea to ensure sufficient fixation by PFA. Fixation was performed in 4% PFA for 20 min. at RT. Dissection was performed in 4% PFA and retinas were incubated in 4% PFA/PBS for 2 h at room temperature followed by washing with PBS and mounting on glass slides.

### Bulk RNA-sequencing analysis

RNA-seq was performed using CD31+ endothelial cells isolated from the retinas of P6 EC-*Foxc1*-KO mice and their respective littermate controls. Retinas pooled from 3 to 6 neonates were digested in collagenase Type I solution (2 mg/mL) for 40 min at 37 °C with gentle agitation. Cells were then filtered through a 70 μm cell strainer and the pellet was resuspended in Buffer 1 (PBS, 0.1% BSA, 2 mM EDTA, pH 7.4). The cell suspension was then incubated with magnetic Dynabeads (Invitrogen) pre-coated with CD31 antibody to isolate the endothelial cell population. After several washes with Buffer 1, RNA was extracted from endothelial cells using RNA STAT solution (Tel-Test) followed by phenol-chloroform treatment. Library construction and sequencing were then performed from 3 biological replicates per experimental group at the Genomics Core facility at the University of Chicago. To generate RNA-sequencing libraries, RNA quality and quantity were determined with the Agilent Bioanalyzer 2100, accepting RNA integrity numbers (RIN) of >7 and quantities of 100 nanograms or more per sample. Directional mRNA libraries were prepared using Illumina TruSeq mRNA Sample Preparation Kits per the manufacturer’s instructions. Briefly, polyadenylated mRNAs were captured from total RNA using oligo-dT selection. Next, samples were converted to cDNA by reverse transcription, and each sample was ligated to Illumina sequencing adapters containing unique barcode sequences. Barcoded samples were then amplified by PCR and the resulting cDNA libraries by quantified using qPCR. Finally, equimolar concentrations of each cDNA library were pooled and sequenced on the Illumina HiSeq2500.

### Transcriptome analysis

The quality of DNA reads, in fastq format, was evaluated using FastQC (https://www.bioinformatics.babraham.ac.uk/projects/fastqc/). Adapters were trimmed with Trim Galore! (https://www.bioinformatics.babraham.ac.uk/projects/trim_galore/),and reads of poor quality or aligning to rRNA sequences were filtered. The cleaned reads were aligned to the *Mus musculus* genome (mm10) using STAR^62^. Read counts for each gene were calculated using htseq-count^63^ in conjunction with a gene annotation file for mm10 obtained from UCSC (University of California Santa Cruz; http://genome.ucsc.edu). Differential expression was determined using DESeq2^64^. The cutoff for determining significantly differentially expressed genes was a FDR-adjusted *p* value less than 0.05.

Pathway enrichment analysis on DEGs was performed using Metascape^65^ on the KEGG, Canonical Pathways, GO, Reactome, and CORUM databases. *P* < 0.05 was considered to be statistically significant.

### Forkhead box C transcription factor binding prediction analysis

Identification of putative FOXC-binding sites was performed as previously described^18^. Briefly, putative FOXC-binding sites were determined first by using the Hypergeometric Optimization of Motif EnRichment (HOMER)^66^ suite of tools to scan the entire Genome Reference Consortium Human Build 37 (GRCh37 or hg19) genome corresponding to the conserved RYMAAYA FOX or RYACACA FOXC-binding motif. The output file was then uploaded to the UCSC genome browser^67^ to identify putative binding sites corresponding to transcriptionally active areas as indicated by histone modification, DNAse sensitivity, and additional transcription factor chromatin immunoprecipitation data as per work reported and summarized in the Encyclopedia of DNA Elements (ENCODE; https://genome.ucsc.edu/ENCODE/). Putative sites in the human genome were then searched against the mm10 mouse genome using the ECR Browser (https://ecrbrowser.dcode.org)^68^ and rVista 2.0 tools to identify conserved and aligned putative binding sites between mouse and human sequences.

### ChIP assay

Human retina microvascular endothelial cells (HRMVECs, Cell Systems ACBRI 181), from a male donor, were cultured from passages 6−8 on gelatin-coated plates using the EGM-2MV culture media (Lonza, CC-3202). The cells were cross-linked with 1% formaldehyde, followed by sonication. The sheared chromatin was immunoprecipitated with Dynabeads (Invitrogen, #10004D) conjugated with anti-FOXC1 antibody (Abcam, ab5079, 2.5 μg/reaction) or control IgG (Thermo Fisher Scientific, #02-6202, 2.5 μg/reaction). DNA extraction and qPCR were performed as previously described^38^ with primers listed in Supplementary Table 1 targeting identified ECRs containing putative binding site sequences shown underlined below. The samples were loaded on agarose gels to confirm the sizes of qPCR products. Images were acquired with a ChemiDoc Imaging System (Bio-Rad).

*SLC3A2 ECR-1.>*hg19 chr11:62618356-62618445

CATGCCTGTAATCCCAGCACTTTGGGAGGCCGAGGCGGGTGGATCACCTGAGGTCAGGAGTTCAAGAGCAGCCTGACCAACATGGAGAAACCCCGTCTCTACTGAAAAAAAAAAAAATATATATATATATATATATACATATACACACACACACACACACACACACACACACAATAATTAGCCAGGCATGGTGGCGCATGCCTGTAATCCCAGCTACTTGGGAGGCTAAGGCAGGAGAATCGCTTGAACCTGGGAGGCGGAGGTTGCGGTGAGCAGAGATCCTGCCATTG

*SLC3A2 ECR-2.>* hg19 chr11:62630045-62630146

GATGAAACCCCCATCTCTACAGGAAAAAAGCCCTTCCTCCCTATCGCCACCCCTCCCCCAAGCCTAAACTACCCCTTCTCACCCACAGTCAACTCCCTGGGGTTTGATTTTATCTAGGTAAATCATCCTGCTATCTCTATTGTCTAGCAGAATACAATTCACAAATAGGACTCAATATACATTTATTAGTTACCTCTATAAGTTTTTTTCAGTTTGCCTCTTTATGTTGGGAATGCTGTAAATTTTTAAATGATCTTTTCTCAGCCAATTCATTGACTGTCCTACCTTACCAAATGATTTTG

*SLC3A2 ECR-3.>* chr11:62647066-62647359

TATTATCTTTGAGCACTGGTCTGAACCCACTTGCTTGCCTTCTCCCAGGGGCTGCCCCGTGCACCCCTTGTTCCTAGGGTGGGGATGGGGTTGGATGCCTGGGGTTGCAGAGGGGACTAGCAGAGGGTGACCCTTTTCTCTATGCTATTACTTCAGATCTTGGCTTTTGCTTTCTTCCAAATACACAGCAATCTTGGCTTCCAGCCTAGGTGGGAAAGGGAGAAAGAACCGATTTCCTCCAGCTCCTCTCTGAAAGTAGCCCAACTGGCACTGTTTAGGAACAAGGAGTTCCTGAGAGTGTTGGCACCCCACGGTGTTGATGTCCGGTAGTTCCCTGCTGTGAACCCTGTCTCTGCCCCACTTCAATCCAAGGACCCTGTGGAGGGAATTCTAGGCTTCTTCAAAGCCTCTGCAGTACCAGCTGCCTCCAAGGCCTAACATAGTGAAGTGATAGGGAGACAGGACCTTGGGGGAGTTTGGGGATAACTGTGGGA

*SLC3A2 ECR-4.>* hg19 chr11:62647682-62647845

GTCTGATTCCTAGCTGCCAAACATCCAGGTGCTAGCAGCTGGGGGTGGGGTAGGATAAGAAGGGGGGTGTCTACCTCAACCTTCCTGCCCAAACCATTCATTTTAAAGTACTGTGAAGAAGGCTGGATTAGTGTTAAATTCAATAAAGTTAACAAAGCAAAGTATTGTAGTGAGTACACAATAAATAGTTGCTGCATGAGGAATTTCTTGAAATGAATTCAAGATGAGTCTTGAAAAACGAGTGGGAGTAAGCCGTGCACGGAAGCAGAGGTCTAGCAGAGGTCTAGCGCCAAGTCCCAGAGGCCTGAGAGGGCAAGCCTTGGTGGGGGAAAAGGCAGAAGTTGTGACTGGCTGCCTCTGAGAG

*SLC3A2 ECR-5*.> chr11:62652633-62652892

CCTCAAGTGATCCACCCGCCTCAGCCTCCCAAAGTGTTGGGATTACAGGCGTGAGCCACTGCGCCTGGCCCCATTCTTTCTTGTGCTAACCTTGAACTCCTCCCTCCCCTCTGCAGGCTCTTGATTGCGGGGACTAACTCCTCCGACCTTCAGCAGATCCTGAGCCTACTCGAATCCAACAAAGACTTGCTGTTGACTAGCTCATACCTGTCTGATTCTGGTTCTACTGGGAGCATACAAAATCCCTAGTCACACAGTATTTGAATGCCACTGGCAATCGCTGGTGCAGCTGGAGTGTGAGTACCATGCTGGTGGGAAAGGGGGCAGATGGGAGAAGAAAGGGTTGTTGGGAGACAGAGGCAGAGGTGGGTTATGGGGCTCACTGGAGTGTCTCTCCCTGTAGTTGTCTCAGGCAAGGCTCCTGACTTCCTTCTTGCCGGCTCAACTTCTCCGACTCTA

*SLC7A5 ECR-1*.> hg19 chr16:87907772-87907940

AGATCGCACCACTGCACTCCAGCCTGGGTGACAAAGCGAGACTCCGTCTCAAAAAAAAAATATATATATATATATATATATATATACACACATATATATACACACATATATACACATATATACACACATATATATACACATATATACACACATATATATACATATATGTATATACACATATATACATATATACGTATATATACACATATATACACATATATATACACACACACACATATATATACACACACATATATATATATAAACATCAGCTGGGCGTGTTGGTGCACCGCCTGTAATGGGAGGCTGAGGCAAGAGAATCGCTTGAAACCAGAAGGCAGAGGTTGCAGTGAACCAAGATTGTGCCAC

*SLC7A5 ECR-2*.> chr16:87886194-87886358

CCGTGTGCTTCAGAAGCCTGAGTGACCCCACACAGATGGCCAGAACGCCTGCCCCAGGAGGCGCTGAGATGTGGGGTCAGGCACGCTCAGACAGGTCTCCAGACACCAGCACGTCCTCCTGGGCCCTGTAAGGCACCCAGCTCTGAGCCTCTGTGTGTGTGTGTGTGTGTGTGTGTGTGTGTGTGTGTGTGTGTGTGTGTTGGGGGTTCTGTTGCAATATATAGGCACGCTGAGCCACTGTGTGTGTGTGCGTGTGTGTGTGTGTTTTGGGGGGTTCTGTTGCAATATGTGCGCACGCTGAGCCACTATTCAGGGATCTGTGAGCTGGTGGGGAAACAGCCACCCCCACAACTTAAAATGAAGTA

*SLC7A5 ECR-3*.> chr16:87874547-87874916

GGCTGCAGCGGGCGGCCTCCCCACGCTTCGTCGGGATGTGGGTGACTCTGGAGGGTCACCTGCCACTCTTTAACTGCCAAGGGGCAGTTAAAGGGAGGGGCAGTCCACTCCCTTTAACTGCCCCTTGATGGCTGGGAGATTTGGGATTCCTGGACACGTCAGGGACTGTATGTCCCCAGACCAAGGGACCCAGACATTCCAGAACCTACCATCCTCCATAGGCAAAGAGGCCGCTGTATAATGCCAGCACAATGTTCCCCACATCCAGTTTGGTGCCTTCAAATGAGAAGTTGGGATCTAGATTGGACACATCACCTGGCAGGGCCAAAGAAAGGAATGCTGGGTTAGAGAGCGCTGAACAGAGGTCATGCTACCAGTTCTAGAATGTTCCTGGAGCAAAATACATAGCAAACAAAAATGCTGGAGTGAAGGCTTTTCACTGTGACAAATGGTATGTACCTGCTGAGCCACATGGAGGGTGCAGGGTTCCAACCACAACCAGGGGGATGTGGCCCTGCCAGAGGCCCTGTGCCCACCTGAGAGGGCACAGGCAGTTCTGACCACTGCCCA

### Cell culture and RNA interference

Pooled HUVECs were purchased from Lonza (CC-2519) and cultured in endothelial basal medium (Lonza CC-3202) containing 5% fetal bovine serum (FBS), hydrocortisone, hFGF-B, VEGF, R3-IGF-1, ascorbic acid, hEGF and GA-1000. Experiments were done using cells with passage numbers less than 7. For *FOXC1* gene knockdown by RNAi, HUVECs were seeded in gelatin-coated 6-well plates and siRNA transfection was performed using the Fugene transfection reagent according to the manufacturer’s instructions. HUVECs were transfected for 48 h with control or *FOXC1* targeting siRNA using a final siRNA concentration of 100 nM (Origene C, SR320173CL). Total RNA (Qiagen, 74034) and cDNA were prepared (Bio-Rad, 1708891) and subjected to real-time PCR (QuantStudio 3, Applied Biosystems) using primers listed in Supplementary Table 2.

### Oxygen-induced retinopathy

Dams and P7 pups were placed in a Plexiglass chamber with an oxygen controller (Pro-Ox 110; Biospherix, Lacona, NY, USA) and exposed to 75% oxygen until P12. Exposure to hyperoxia was continuous, with brief interruptions only for animal care. Dams and fosters were rotated every 24 h from hyperoxia to room air to prevent excessive oxygen toxicity. Following exposure to hyperoxia, pups were administered 100 µg of tamoxifen diluted in corn oil by subcutaneous injection daily from P13 to P17. Mice were euthanized and eyes were collected at P18 for fixation, then retinas were dissected and immunostained for analysis of the retina vasculature. Cre-negative, tamoxifen-treated littermates were utilized as controls during analysis.

OIR retinal vascular leakage was assessed in P18 EC-*Foxc1*-KO and littermate control pups exposed to OIR conditions as described above. Briefly after exposure to OIR, the pups were weighed (6.5−9.3 g) and combined injections of 10 kDa-FITC-dextran, 100 mg/mL and fluorescent Alexa568-conjugated IB4 (5 mg/kg) were injected retro-orbitally as described in the previous section.

### Imaging

Imaging was performed using a Zeiss AxioVision inverted fluorescence microscope using 2.5X, 10X, 20X, and 40X objectives and Zeiss AxioVision software, Nikon A1 or AXR Confocal Laser Microscope System using 10X, 20X or 40X objectives, Nikon Ti2 Widefield using 4X or 10X objective and NIS-Elements software and AMG EVOS fluorescence microscope using 4X or 10X objectives. 3D reconstructed images were generated using the Volume Viewer plugin in Fiji software. Fiji/ImageJ, Adobe Photoshop and Adobe Illustrator software were used for image acquisition and processing.

### Western blot analysis

Pooled retina tissue from EC-*Foxc1*-KO mice and littermate controls were homogenized in RIPA buffer supplemented with a protease inhibitor cocktail (Sigma-Aldrich, 11836170001). Following centrifugation, the supernatant was isolated and protein concentration was determined with a Pierce 660 nm Protein Assay (Thermo Fisher, 22662). 30 μg of protein lysate was loaded per lane onto a 4−20% Mini-PROTEAN TGX Stain-Free gel (Bio-Rad, 4568094) for SDS-PAGE. Proteins were then transferred onto 0.45 μm nitrocellulose membranes. Following protein transfer, the membranes were incubated in 5% non-fat milk (NFM) for 1 h at room temperature on a rocking plate prior to incubation at 4 °C overnight on a rocking plate with primary antibodies against FOXC1 (Cell Signaling Technologies, 8758 S, 1:500), LAT1 (Santa Cruz Biotechnologies, sc-374232, 1:500) and β-actin (Proteintech, 66009-1-Ig, 1:10000). The next day, membranes were washed in 1X TBST, then incubated with Goat anti-Rabbit IgG, HRP-conjugate (Millipore, 12-348; 1:2000) or Donkey anti-Mouse IgG, HRP-conjugate (Invitrogen, A16011, 1:2000) diluted in 5% NFM at room temperature on a rocking plate for 2 h. Protein bands were visualized with SuperSignal West Pico Plus Chemiluminescent substrate (Thermo Fisher, 34577), and protein band images were acquired using an Azure Biosystems c600 imaging system. Uncropped blots are provided in the Source Data file.

### Quantification

Quantification of retina vessel outgrowth length in neonatal mice was completed by measuring the distance from the center of the optic nerve head to the angiogenic front from 6 to 8 leaflets per individual using Fiji software. For quantification of vascular density and branching index in neonatal mice, 6−8 10X high-power field (HPF) images were acquired from the rear capillary plexus per individual and 3−10 10X HPF images were acquired from the arteriole and venule fronts per individual, which were analyzed using AngioTool per the developers’ recommended guidelines^69^. For filopodia analysis, the total filopodia number was counted along the angiogenic front from 4 40X HPF images per individual. The length of the angiogenic front in each field was then measured to determine the total filopodia number per 1000 µm vessel length using Fiji software. Endothelial proliferation was measured by counting the number of phospho-histone H3 (pHH3)/Isolectin-B4 (IB4)-positive cells per total vascular area from 8 10X HPF per individual using Fiji. Endothelial apoptosis was measured by counting the number of cleaved caspase 3/IB4 positive cells per total vascular area from 7 to 8 10X HPF per individual using Fiji. Number of FOXC1-positive endothelial cells and pericytes were calculated per vascular area manually based on the distinct morphology of endothelial cells and pericytes using Fiji. Elongated endothelial cells and densely stained spherical pericytes were identified based on the IB4 staining and PDGFRβ staining respectively in 3−6 vascularized fields per sample^70^. The fluorescent intensity of PDGFRβ was measured to determine the percent mean fluorescent intensity per vascular area using Fiji from 3 to 6 fields per individual. Vascular leakage was determined as dextran positive area subtracted from the IB4 positive area and presented as percentage per vascular area. Absolute intensity of pS6 in IB4 positive area was quantified in retinal endothelial cells using Fiji and expressed as % relative to controls. For ChIP studies, qPCR was performed from 5 to 8 replicates per each ECR. The vascular density of P18 mice kept under normoxia conditions was measured from individual retina leaflets from each mouse using Fiji. For OIR analysis, stitched images of the entire retina vasculature were generated and the avascular area was measured from both eyes per individual using Fiji. For quantification of the neovascular tuft area, the SWIFT_NV ImageJ macro was utilized following the authors’ guidelines^35^. For quantification of pericyte coverage area in Pericyte-*Foxc1*-KO and littermate control pups, the surface area positive for pericyte marker immunolabeling (PDGFRβ) was divided by the surface area positive for the endothelial maker (IB4) in 2−5 fields per individual and presented as %.

### Statistics

Statistical analysis was performed on averaged measurements from individual mice or each biological replicate using GraphPad Prism v10. *P* values were obtained by performing a 2-tailed, unpaired Student’s *t* test or one-way ANOVA with Dunnett’s or Tukey’s multiple comparisons test where indicated. Data are presented as mean ± standard deviation (SD) of representative experiments from at least three biological replicates. *P* values less than 0.05 were considered statistically significant.

### Reporting summary

Further information on research design is available in the Nature Portfolio Reporting Summary linked to this article.

### Supplementary information

Supplementary Information

Description of Additional Supplementary Files

Supplementary Data 1

Reporting Summary

### Source data

Source Data

## Data Availability

The RNA-seq datasets have been deposited in NCBI’s Gene Expression Omnibus under the accession number GSE262908. All data needed to evaluate the conclusions in the paper are present in the paper and/or the Supplementary Materials. Additional data related to this paper may be requested from the authors. Source data are provided with this paper.
