# Supplementary material for: FOXC1 regulates endothelial CD98 (LAT1/4F2hc) expression in retinal angiogenesis and blood-retina barrier formation
Source: Nat Commun. 2024 May 16;15:4097. doi: 10.1038/s41467-024-48134-2 (PMC11099035; doi:10.1038/s41467-024-48134-2)
Supplement: Supplementary file 1 — Supplementary Information [file 41467_2024_48134_MOESM1_ESM.pdf]

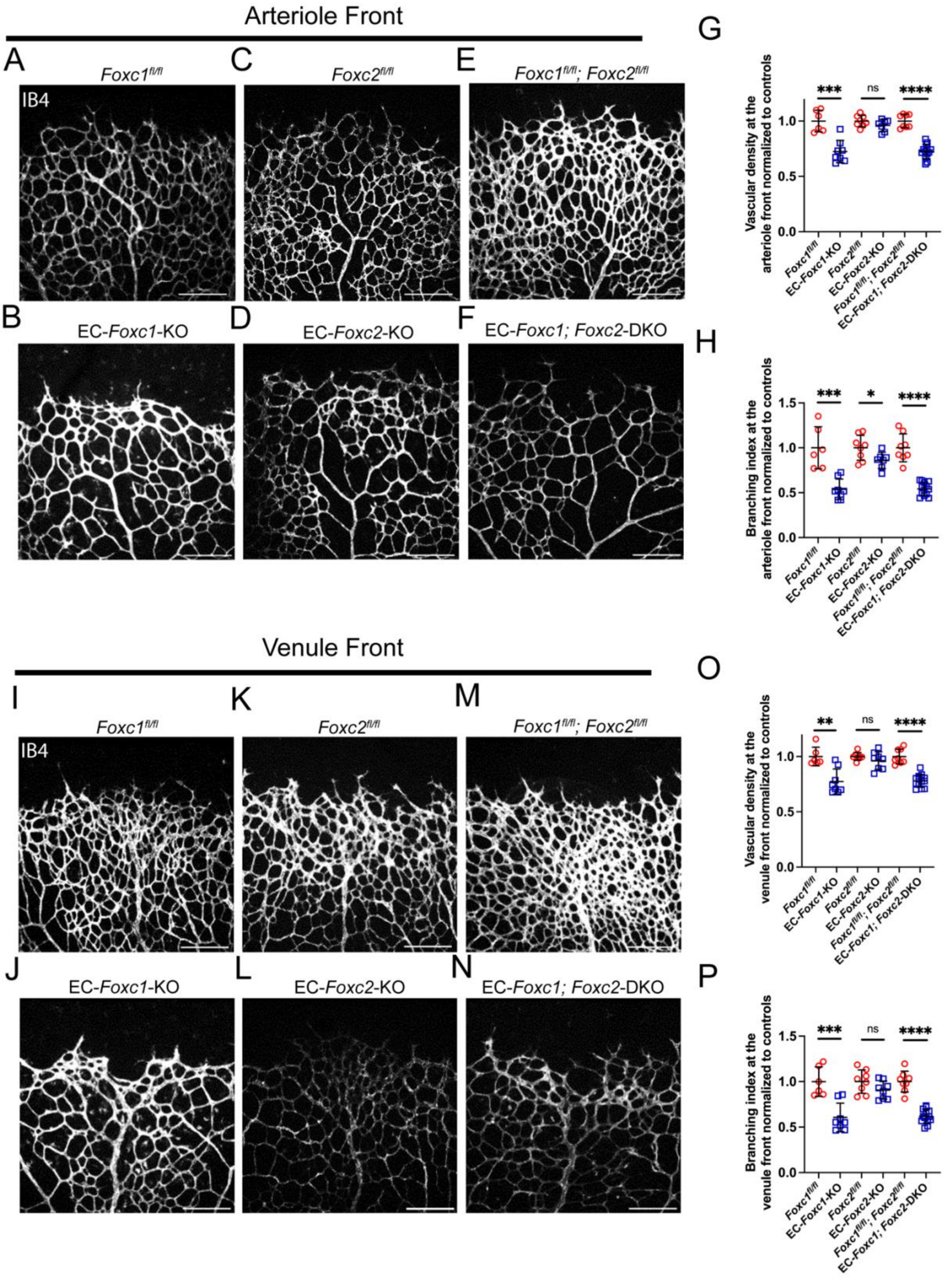

Arteriole Front
A
Foxc1fl/fl
IB4
C
Foxc2fl/fl
E
Foxc1fl/fl; Foxc2fl/fl
B
EC-Foxc1-KO
D
EC-Foxc2-KO
F
EC-Foxc1; Foxc2-DKO
G
Vascular density at the arteriole front normalized to controls
***
ns
****
1.0
0.5
0.0
H
Branching index at the arteriole front normalized to controls
***
*
****
1.5
1.0
0.5
0.0
Foxc1fl/fl
EC-Foxc1-KO
Foxc2fl/fl
EC-Foxc2-KO
Foxc1fl/fl; Foxc2fl/fl
EC-Foxc1; Foxc2-DKO
Venule Front
I
Foxc1fl/fl
IB4
K
Foxc2fl/fl
M
Foxc1fl/fl; Foxc2fl/fl
J
EC-Foxc1-KO
L
EC-Foxc2-KO
N
EC-Foxc1; Foxc2-DKO
O
Vascular density at the venule front normalized to controls
**
ns
****
P
Branching index at the venule front normalized to controls
***
ns
****

Supplementary Fig. 1: Endothelial-specific deletion of *Foxc1* impairs vascular development at the angiogenic front. (**A – F**) Representative images of IB4 staining of the retina vascular arteriole front in P6 littermate control (**A**, **C**, **E**) or EC-*Foxc1*-KO (**B**) EC-*Foxc2*-KO (**D**), or EC-*Foxc1; Foxc2*-DKO (**F**) mice. Scale bars are 200 µm. (**G**) Quantification of vascular density per 10X HPF in P6 EC-*Foxc1*-KO (*n* = 8), EC-*Foxc2*-KO (*n* = 7), or EC-*Foxc1; Foxc2*-DKO (*n* = 13) mice compared to $Foxc1^{fl/fl}$ (n = 6), $Foxc2^{fl/fl}$ (n = 8) and $Foxc1^{fl/fl}$; $Foxc2^{fl/fl}$ (n = 8) mice. Data are mean ± SD. ns, not significant; *** p = 0.0002, **** p < 0.0001. Two-tailed unpaired t-test. (**H**) Quantification of branching index per 10X HPF at the arteriole front in P6 EC-*Foxc1*-KO (*n* = 8), EC-*Foxc2*-KO (*n* = 7), or EC-*Foxc1; Foxc2*-DKO (*n* = 13) mice compared to $Foxc1^{fl/fl}$ (n = 6), $Foxc2^{fl/fl}$ (n = 8) and $Foxc1^{fl/fl}$; $Foxc2^{fl/fl}$ (n = 8) mice. Data are mean ± SD. * p = 0.0395, *** p = 0.0004, **** p < 0.0001. Two-tailed unpaired t-test. (**I – N**) Representative images of IB4 staining of the retina vascular venule front in P6 littermate control (**I**, **K**, **M**) or EC-*Foxc1*-KO (**J**) EC-*Foxc2*-KO (**L**), or EC-*Foxc1; Foxc2*-DKO (**N**) mice. Scale bars are 200 µm. (**O**) Quantification of vascular density per 10X HPF in P6 EC-*Foxc1*-KO (*n* = 8), EC-*Foxc2*-KO (*n* = 8), or EC-*Foxc1; Foxc2*-DKO (*n* = 13) mice compared to $Foxc1^{fl/fl}$ (n = 6), $Foxc2^{fl/fl}$ (n = 8) and $Foxc1^{fl/fl}$; $Foxc2^{fl/fl}$ (n = 8) mice. Data are mean ± SD. ns, not significant; ** p = 0.0015, **** p < 0.0001. Two-tailed unpaired t-test. (P) Quantification of branching index per 10X HPF at the venule front in P6 EC-*Foxc1*-KO (*n* = 8), EC-*Foxc2*-KO (*n* = 8), or EC-*Foxc1; Foxc2*-DKO (*n* = 13) mice compared to $Foxc1^{fl/fl}$ (n = 6), $Foxc2^{fl/fl}$ (n = 8) and $Foxc1^{fl/fl}$; $Foxc2^{fl/fl}$ (n = 8) mice. Data are mean ± SD. ns, not significant; ***p = 0.0006; **** p < 0.0001. Two-tailed, unpaired t-test. Source data are provided as a Source Data file.

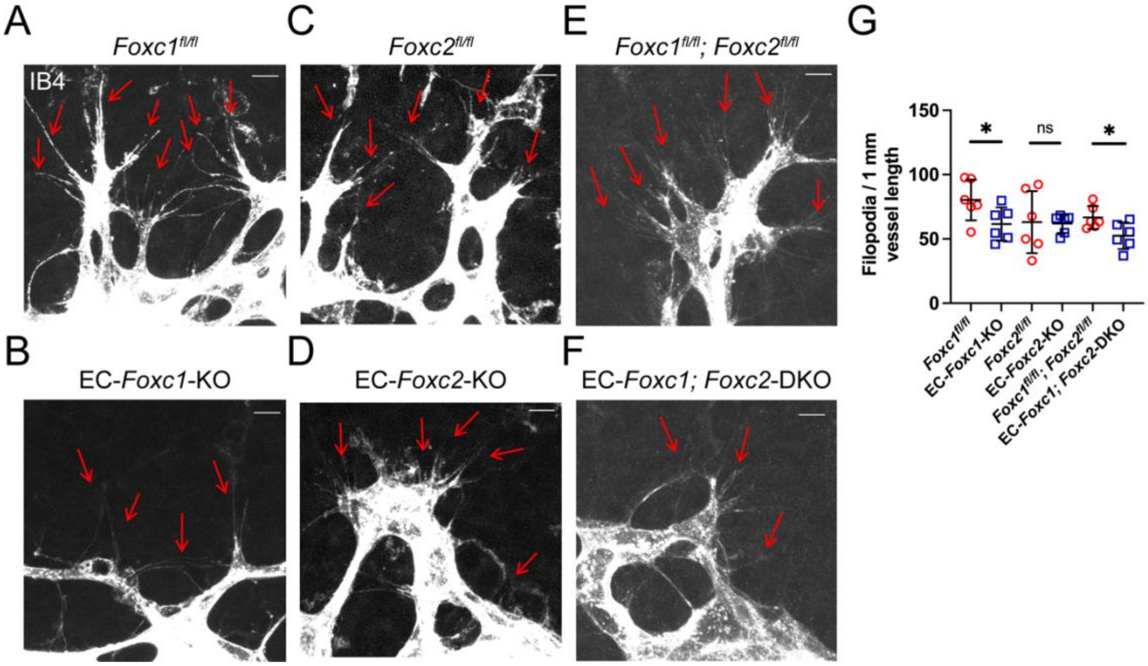

Supplementary Fig. 2: Endothelial-specific deletion of *Foxc1* results in loss of filopodia formation at the angiogenic front. (**A-F**) Representative images of IB4 staining of the angiogenic front in P6 littermate control (**A, C, E**) or EC-*Foxc1*-KO (**B**), EC-*Foxc2*-KO (**D**), or EC-*Foxc1; Foxc2*-DKO (**F**) mice. Red arrowheads denote filopodia present in the endothelium along the angiogenic front. Scale bars are 10 μm. (**G**) Quantification of filopodia number per 1000 μm vessel length at the angiogenic front in P6 EC-*Foxc1*-KO (n = 6), EC-*Foxc2*-KO (n = 6), or EC-*Foxc1; Foxc2*-DKO (n = 6) mice compared to *Foxc1*$^{fl/fl}$ (n = 6), *Foxc2*$^{fl/fl}$ (n = 6) and *Foxc1*$^{fl/fl}$*; Foxc2*$^{fl/fl}$ (n = 6) mice. Data are mean ± SD. ns, not significant; * p = 0.0491 (*Foxc1*$^{fl/fl}$ vs. EC-*Foxc1*-KO) and 0.0286 (*Foxc1*$^{fl/fl}$; *Foxc2*$^{fl/fl}$ vs. EC-*Foxc1; Foxc2*-DKO). Student's two-tailed, unpaired t-test. Source data are provided as a Source Data file.

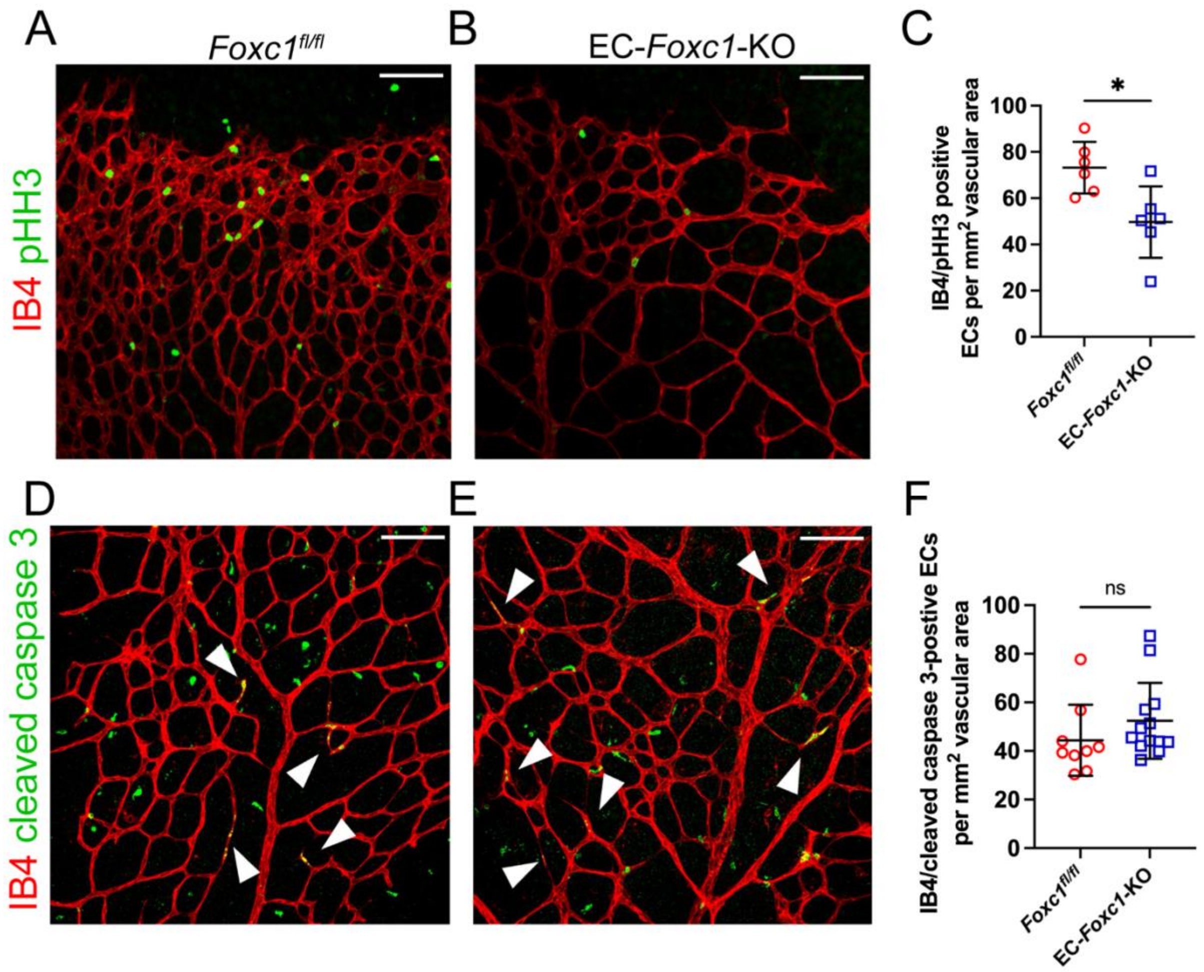

Supplementary Fig. 3: Endothelial proliferation is decreased in EC-*Foxc1*-KO mice. (**A-B**) Representative images of IB4 and pHH3 staining in the retina vasculature of a P6 littermate control (**A**) and EC-*Foxc1*-KO (**B**) individuals. Scale bars are 100 µm. (**C**) Quantification of the number of pHH3+ ECs per mm^2 vascular area in P6 EC-*Foxc1*-KO (n = 6) mice compared to littermate controls (n = 6). Data are mean ± SD. * p = 0.0128. Student's two-tailed, unpaired t-test. (**D-E**) Representative images of IB4 and cleaved caspase 3 staining in the retina vasculature of a P6 littermate control (**D**) and EC-*Foxc1*-KO (**E**) individuals. Arrowheads denote IB4+ and cleaved caspase 3+ ECs in regressing vessels. Scale bars are 100 µm. (**F**) Quantification of the number of cleaved caspase 3- positive ECs per mm^2 vascular area in P6 EC-*Foxc1*-KO (n = 13) mice compared to littermate controls (n = 9). Data are mean ± SD. ns, not significant. Student's two-tailed, unpaired t-test. Source data are provided as a Source Data file.

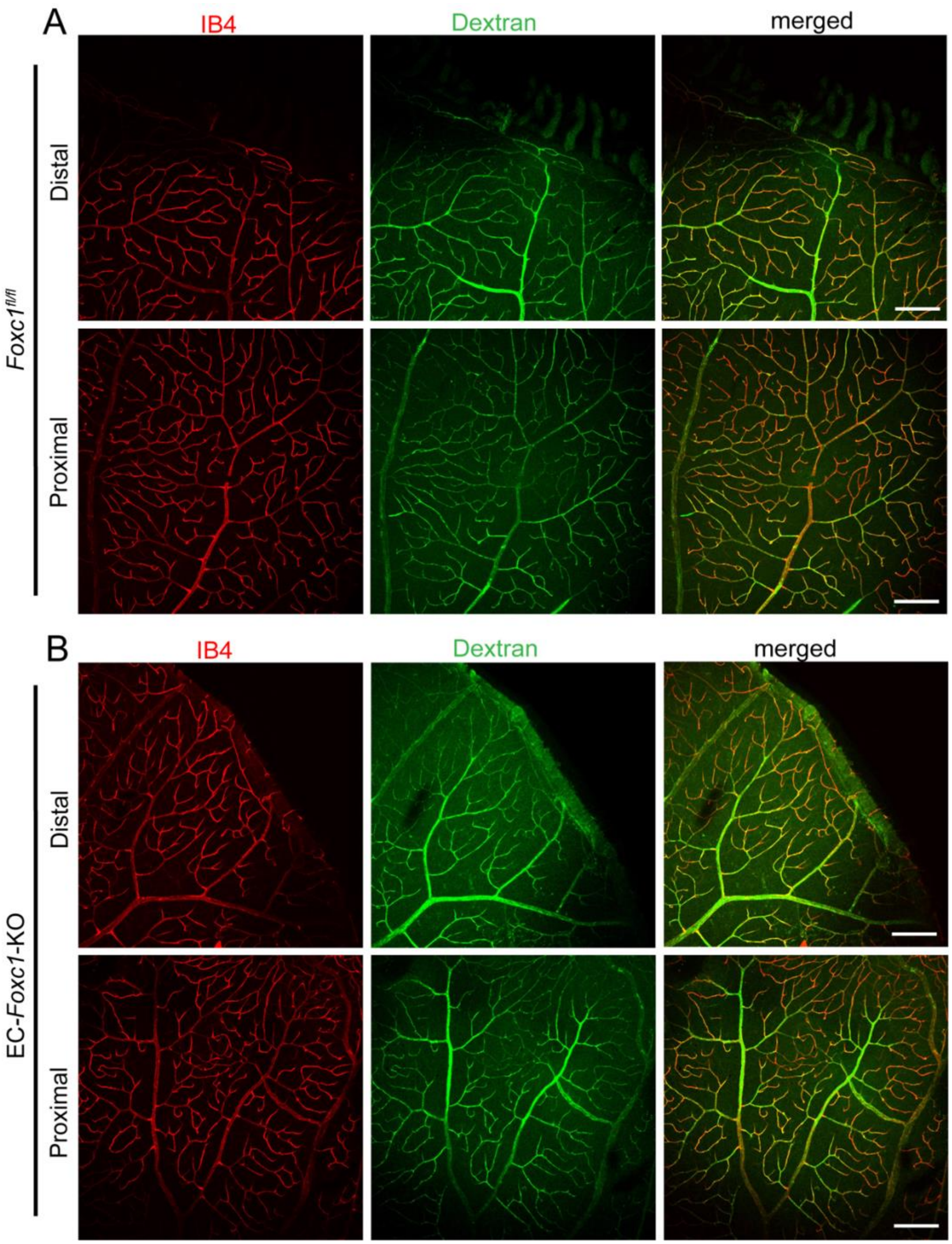

Supplementary Fig. 4: Late postnatal endothelial-specific deletion of *Foxc1* does not alter the vascular permeability of the retina vasculature. (A-B) Representative images of retina vessel leakage in the superficial plexus as assessed by retroorbital injections of FITC-dextran and IB4-568 in P21 littermate control **(A)** and EC-*Foxc1*-KO **(B)** individuals. Analysis was carried out in 4 EC-*Foxc1*-KO and 5 littermate control mice. Scale bars are 200 μm.

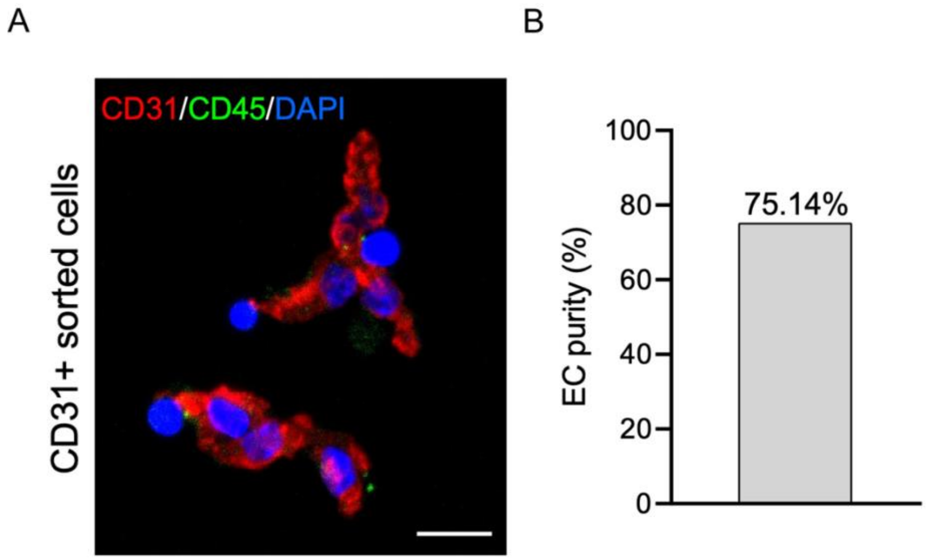

Supplementary Fig. 5: Assessment of purity of isolated endothelial cells for transcriptomic analyses. CD31+ cells were sorted from neonatal retina at P6 by using CD31 antibody and Dynabeads. Cells were stained with CD31 (red) and CD45 (green) as well as DAPI **(A)** to determine the purity of CD45-CD31+ endothelial cells (ECs) (percentage of the number of CD45-CD31+ ECs in relation to the total cell number) as shown in **B**. Scale bar = 10 µm. Source data are provided as a Source Data file.

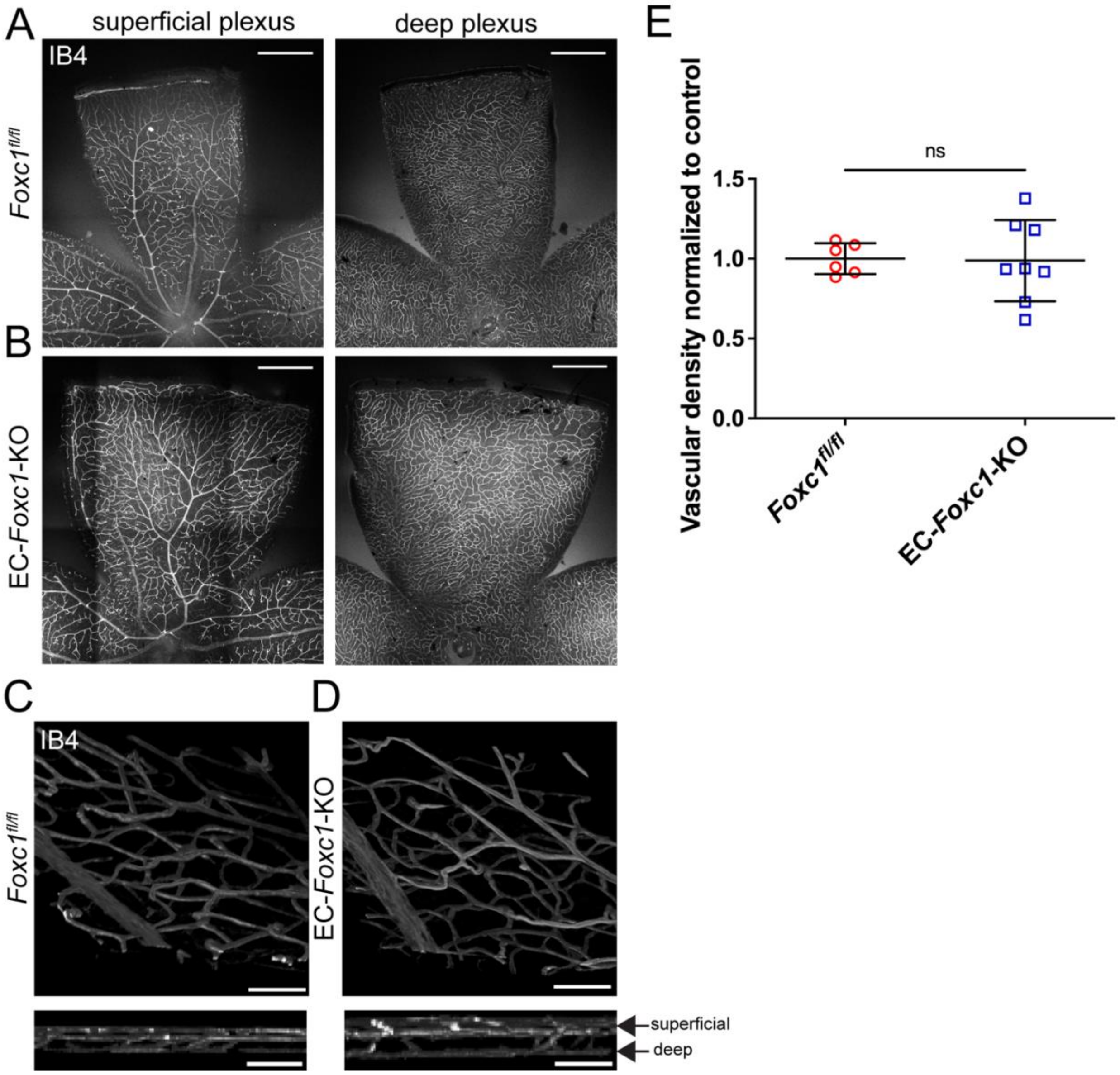

Supplementary Fig. 6: Endothelial FOXC1 is dispensable during late stages of postnatal retinal angiogenesis. (**A, B**) Representative images of IB4 staining of the superficial **(A)** and deep **(B)** retina vasculature in P18 littermate control (**B**) and EC-*Foxc1*-KO (**C**) mice exposed to normoxia conditions shown in **Figure 8A**. Scale bars are 500 μm. (**C, D**) 3D reconstructed images and cross-sections of IB4-stained retinal vasculature of littermate control (**C**) and EC-*Foxc1*-KO mice (**D**). Scale bars are 50 μm. (**E**) Quantification of vascular density in retina leaflets of P18 control (*n* = 6) and EC-*Foxc1*-KO (*n* = 8) mice. Data are mean ± SD. ns, not significant. Student's two-tailed, unpaired t-test. Source data are provided as a Source Data file.

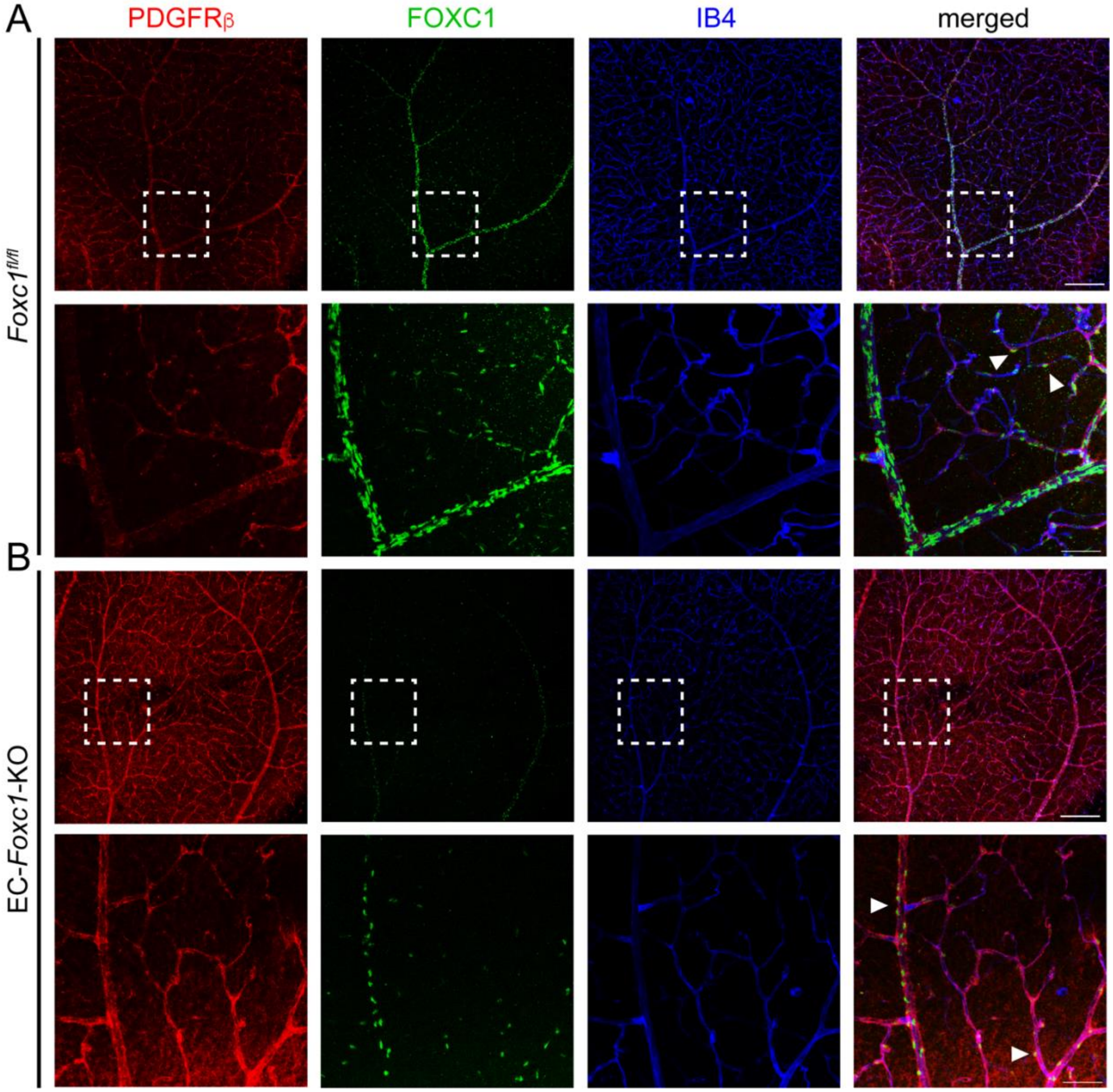

Supplementary Fig. 7: Characterization of FOXC1 expression during late stages of postnatal retina angiogenesis. (**A, B**) Representative images of PDGFRβ, FOXC1, and IB4 stained retina vasculature in P18 littermate control (**A**) and EC-*Foxc1*-KO (**B**) mice. White boxes denote magnified regions depicted in lower panels. White arrowheads denote PDGFRβ+ and FOXC1+ pericytes recruited to the abluminal surface of the retina endothelium. Scale bars are 200 and 50 μm respectively.

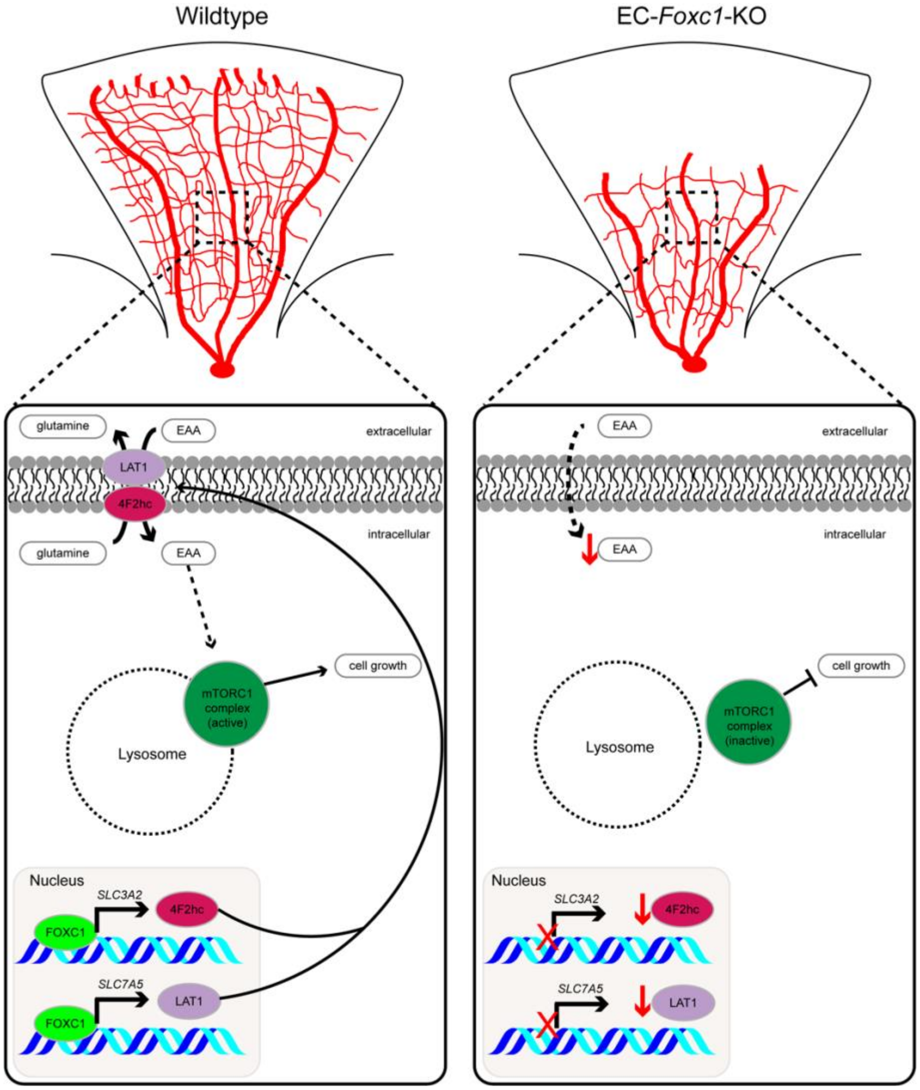

Supplementary Fig. 8: Endothelial-derived FOXC1 controls physiological retina angiogenesis by regulation of 4F2hc and LAT1 expression and mTORC1 activation. In wildtype mice, FOXC1 regulates expression of *SLC3A2*, encoding 4F2hc, and *SLC7A5*, encoding LAT1, and the protein products assemble into a heterodimeric, bidirectional amino acid transporter at the plasma membrane. Exchange of intracellular glutamine for essential amino acids (EAA) then promotes activation of mTORC1, localized to cellular lysosomes, and downstream anabolic pathways promoting cell growth. In contrast, endothelial-specific deletion of *Foxc1* results in significantly reduced *Slc3a2* and *Slc7a5*, which results in markedly reduced mTORC1 activity, thus impairing physiological angiogenesis.

Supplementary Table 1: Primers used for ChIP analysis

Evolutionary Conserved Regions (ECRs)	Forward	Reverse
SLC3A2 ECR-1	GGAGAAACCCCGTCTCTACTG	GCCTTAGCCTCCCAAGTAGC
SLC3A2 ECR-2	ACTCCCTGGGGTTTGATTTT	TTCCCAACATAAAGAGGCAAA
SLC3A2 ECR-3	AGGTGGGAAAGGGAGAAAGA	CAGAGACAGGGTTCACAGCA
SLC3A2 ECR-4	CTGCCCAAACCATTCATTTT	CATTTCAAGAAATTCCTCATGC
SLC3A2 ECR-5	TCTTGATTGCGGGGACTAAC	GGGATTTTGTATGCTCCCAGT
SLC7A5 ECR-1	AGCGAGACTCCGTCTCAAAA	CACGCCCAGCTGATGTTTAT
SLC7A5 ECR-2	AGGTCTCCAGACACCAGCAC	GTGGCTCAGCGTGCCTAT
SLC7A5 ECR-3	GGGTTAGAGAGCGCTGAACA	GAAAAGCCTTCACTCCAGCA

Supplementary Table 2: Primers used for real-time PCR

Gene	Forward	Reverse
FOXC1	TCACAGAGGATCGGCTTGAAC	CGTGCGGTACAGAGACTGG
SLC3A2	GGACTAACTCCTCCGACCT	GCCAATCTCATCCCCGTA
SLC7A5	TTATCGGCTCGGGCATCTTC	TTGGACACATCACCCTTCCCA
PPIA	CCTAAAGCATACGGGTCCTG	TTTCACTTTGCCAAACACCA

Supplementary method:

Assessment of purity of isolated endothelial cells for transcriptomic analyses

Eyes from 7 pups at P6 were collected. The retinae were dissected and processed upon dissociating into single cell suspension. Endothelial cells (ECs) were isolated by using CD31 antibody (BD #553369, 1:50) and Invitrogen™ Dynabeads™ FlowComp™ Flexi Kit (Invitrogen #11061D) according to the manufacturer's instructions. After releasing the CD31+ cells from Dynabeads, the cells were stained with Alexa Fluor-647 conjugated Goat anti Rat IgG (Invitrogen #A21247, 1:500), followed by rat serum blocking and CD45-FITC (Biolegend #103108, 1:100) incubation. The cells were then fixed in 4% PFA and stained with DAPI (Sigma, D9542-5MG, 10 μg/mL), followed by mounting on a glass slide for further confocal imaging. More than 350 total cells were imaged under 20x magnification and counted. The EC purity was determined by calculating the percentage of the number of CD45-CD31+ cells in relation to the total cell number.
