# Supplementary material for: FOXC1 regulates endothelial CD98 (LAT1/4F2hc) expression in retinal angiogenesis and blood-retina barrier formation
Source: Nat Commun. 2024 May 16;15:4097. doi: 10.1038/s41467-024-48134-2 (PMC11099035; doi:10.1038/s41467-024-48134-2)
Supplement: Supplementary file 2 — Description of Additional Supplementary Files [file 41467_2024_48134_MOESM2_ESM.pdf]

Description of Additional Supplementary Files

Supplementary Data 1 - Differentially expressed genes (DEGs) in P6 retina endothelial cells from bulk RNA-seq analysis of EC-Foxc1-KO mice and littermate controls.
