# Supplementary material for: FOXC1 regulates endothelial CD98 (LAT1/4F2hc) expression in retinal angiogenesis and blood-retina barrier formation
Source: Nat Commun. 2024 May 16;15:4097. doi: 10.1038/s41467-024-48134-2 (PMC11099035; doi:10.1038/s41467-024-48134-2)
Supplement: Supplementary file 4 — Reporting Summary [file 41467_2024_48134_MOESM4_ESM.pdf]

nature portfolio

Corresponding author(s): Tsutomu Kume

Last updated by author(s): 3/29/2024

Reporting Summary

Nature Portfolio wishes to improve the reproducibility of the work that we publish. This form provides structure for consistency and transparency in reporting. For further information on Nature Portfolio policies, see our Editorial Policies and the Editorial Policy Checklist.

Statistics

For all statistical analyses, confirm that the following items are present in the figure legend, table legend, main text, or Methods section.

n/a	Confirmed	
☐	☒	The exact sample size (*n*) for each experimental group/condition, given as a discrete number and unit of measurement
☐	☒	A statement on whether measurements were taken from distinct samples or whether the same sample was measured repeatedly
☐	☒	The statistical test(s) used AND whether they are one- or two-sided<br>*Only common tests should be described solely by name; describe more complex techniques in the Methods section.*
☒	☐	A description of all covariates tested
☒	☐	A description of any assumptions or corrections, such as tests of normality and adjustment for multiple comparisons
☐	☒	A full description of the statistical parameters including central tendency (e.g. means) or other basic estimates (e.g. regression coefficient) AND variation (e.g. standard deviation) or associated estimates of uncertainty (e.g. confidence intervals)
☐	☒	For null hypothesis testing, the test statistic (e.g. *F*, *t*, *r*) with confidence intervals, effect sizes, degrees of freedom and *P* value noted<br>*Give P values as exact values whenever suitable.*
☒	☐	For Bayesian analysis, information on the choice of priors and Markov chain Monte Carlo settings
☒	☐	For hierarchical and complex designs, identification of the appropriate level for tests and full reporting of outcomes
☒	☐	Estimates of effect sizes (e.g. Cohen's *d*, Pearson's *r*), indicating how they were calculated

Our web collection on statistics for biologists contains articles on many of the points above.

Software and code

Policy information about availability of computer code

Data collection	Imaging: Nikon A1 or Nikon AXR confocal microscope, Zeiss AxioVision inverted fluorescence microscope, Nikon Ti2 Widefield, AMG EVOS fluorescence microscope<br>RNA-seq: Illumina HiSeq2500<br>ChIP assay: ChemiDoc Imaging System (Bio-Rad)<br>Western-blot: Azure Biosystems c600<br>qRT-PCR: QuantStudio 3 (Applied Biosystems)
Data analysis	Graphs: GraphPad Prism (v10)<br>Image analysis: ImageJ/FIJI (version: 2.14.0/1.54f), NIS Elements Viewer (version 4.11.0), AngioTool (version 0.5a), Adobe Photoshop (25.4.0 release), Adobe Illustrator (28.3)<br>RNA-seq: FastQC, Trim Galore!, STAR, htseq-count, DESeq2, Metascape<br>Forkhead box C binding prediction: HOMER, rVista 2.0

For manuscripts utilizing custom algorithms or software that are central to the research but not yet described in published literature, software must be made available to editors and reviewers. We strongly encourage code deposition in a community repository (e.g. GitHub). See the Nature Portfolio guidelines for submitting code & software for further information.

Data

Policy information about availability of data

All manuscripts must include a data availability statement. This statement should provide the following information, where applicable:

- Accession codes, unique identifiers, or web links for publicly available datasets
- A description of any restrictions on data availability
- For clinical datasets or third party data, please ensure that the statement adheres to our policy

RNA-seq datasets have been deposited in NCBI's Gene Expression Omnibus under the accession number GSE262908. All data needed to evaluate the conclusions in the paper are present in the paper and/or the Supplementary Materials. Additional data related to this paper may be requested from the authors.

Research involving human participants, their data, or biological material

Policy information about studies with human participants or human data. See also policy information about sex, gender (identity/presentation), and sexual orientation and race, ethnicity and racism.

Reporting on sex and gender	N/A
Reporting on race, ethnicity, or other socially relevant groupings	N/A
Population characteristics	N/A
Recruitment	N/A
Ethics oversight	N/A

Note that full information on the approval of the study protocol must also be provided in the manuscript.

Field-specific reporting

Please select the one below that is the best fit for your research. If you are not sure, read the appropriate sections before making your selection.

☒ Life sciences ☐ Behavioural & social sciences ☐ Ecological, evolutionary & environmental sciences

For a reference copy of the document with all sections, see nature.com/documents/nr-reporting-summary-flat.pdf

Life sciences study design

All studies must disclose on these points even when the disclosure is negative.

Sample size	No statistical methods were used to predetermine the sample size. Sample size determination is based on the pilot experiments and previous experiments to obtain reproducibility and significance. For in vivo experiments the group sizes were determined empirically, based on our prior knowledge of intra-group variation associated with the control and mutant groups. We usually use at least 3 mice per group. Likewise, in vitro experiments employed at least 3 biological replicates. Sample sizes for each group is indicated in the figure legend.
Data exclusions	Mice with unexpected death were excluded from the analysis. After checking the data quality, all data were included.
Replication	All experiments were reproduced in multiple independent experiments. For each panel, the number of independent samples is indicated in the figure legend.
Randomization	No statistical methods were used for randomization. For mouse experiments, genotype for each mouse was determined after tamoxifen treatment and based on the requisite genotype, mice were separated to control or mutant groups for subsequent experiments.
Blinding	Investigators were not blinded for sample allocation due to practical reasons. Mice were selected based on their genotypes and cells were selected based on the treatment received.

Reporting for specific materials, systems and methods

We require information from authors about some types of materials, experimental systems and methods used in many studies. Here, indicate whether each material, system or method listed is relevant to your study. If you are not sure if a list item applies to your research, read the appropriate section before selecting a response.

Materials & experimental systems

n/a	Involved in the study
☐	☒ Antibodies
☐	☒ Eukaryotic cell lines
☒	☐ Palaeontology and archaeology
☐	☒ Animals and other organisms
☒	☐ Clinical data
☒	☐ Dual use research of concern
☒	☐ Plants

Methods

n/a	Involved in the study
☒	☐ ChIP-seq
☒	☐ Flow cytometry
☒	☐ MRI-based neuroimaging

Antibodies

Antibodies used

Primary antibodies used for immunostaining analysis:

Isolectin B4 Alexa Fluor 488 conjugate (Thermo Fisher Scientific, I21411, 1:100)
Isolectin B4 Alexa Fluor 568 conjugate (Thermo Fisher Scientific, I21412, 1:100)
Isolectin B4 Alexa Fluor 647 conjugate (Thermo Fisher Scientific, I32450, 1:100)
FoxC1 (D8A6) (Rabbit mAb, Cell Signaling Technologies, 8758S, 1:50)
FoxC2 (Rat, Kind gift from Dr. N Miura; Miura et al., 1997, Genomics, 1:200)
Phospho-Histone H3 (S10) (pRabbit, Abcam, ab5176, 1:100)
Active caspase 3 (pRabbit, R&D, AF835, 1:100)
Phospho-S6 ribosomal protein (Ser235/236) (Rabbit, Cell Signaling Technologies, 2211S, 1:100/1:500)
CD140b (PDGFRB) (APB5)(mRat, Thermo Fisher Scientific, 14-1402-81, 1:50)

Secondary antibodies used for immunostaining analysis:

Alexa 488-conjugated (Donkey anti-rabbit, Thermo Fisher Scientific, A-21206, 1:500)
Alexa 488-conjugated (Donkey anti-rat, Thermo Fisher Scientific, A-21208, 1:500)
Alexa 568-conjugated (Goat anti-rat, Thermo Fisher Scientific, A-11077, 1:500)
Alexa 568-conjugated (Donkey anti-rat, Abcam, ab175475, 1:500)

Primary antibodies used for western blot analysis:

FoxC1 (D8A6) (Rabbit mAb, Cell Signaling Technologies, 8758S, 1:500)
LAT1 (D-10) (mMouse, Santa Cruz Biotechnologies, sc-374232, 1:500)
Beta-actin (Clone no.2D4H5, mMouse, Proteintech, 66009-1-Ig, 1:10000)

Secondary antibodies used for western blot analysis:
Goat anti-Rabbit IgG, HRP-conjugate (Millipore, 12-348, 1:2000)
Donkey anti-Mouse IgG, HRP-conjugate (Invitrogen, A16011, 1:2000)

Antibodies used for Dynabead coating for isolation of retina endothelial cells
PECAM-1/CD31 (Rat anti-mouse, BD Pharmingen, 553370, 1:50)

Antibodies used for ChIP assay:

FoxC1 (pGoat, Abcam, ab5079, 2.5 microgram/reaction)
Control IgG (Goat, Thermo Fisher Scientific, #02-6202, 2.5 microgram/reaction)

Antibodies used for assessment of purity of isolated retina endothelial cells for transcriptomic analyses
CD31 (Rat, BD #553369, 1:50)
CD45-FITC (30-F11) (mRat, Biolegend #103108, 1:100)
Alexa Fluor-647 conjugated Goat anti Rat IgG (Invitrogen #A21247, 1:500)

Validation

All the antibodies (except anti-FoxC2) used in this manuscript were acquired from the indicated commercial vendors. All antibodies were used in multiple experiments, and target protein was detected at the expected molecular weight, tissue expression pattern and subcellular organization. Antibodies have been validated by the respective manufacturers as stated in their website:

Immunostaining was performed with following antibodies:
Isolectin B4 Alexa Fluor 488 conjugate (https://www.thermofisher.com/order/catalog/product/I21411)
Isolectin B4 Alexa Fluor 568 conjugate (https://www.thermofisher.com/order/catalog/product/I21412)
Isolectin B4 Alexa Fluor 647 conjugate (https://www.thermofisher.com/order/catalog/product/I32450)
FoxC1 (D8A6) (https://www.cellsignal.com/products/primary-antibodies/foxc1-d8a6-rabbit-mab/8758)
Phospho-Histone H3 (S10) (https://www.abcam.com/products/primary-antibodies/histone-h3-phospho-s10-antibody-ab5176.html)
Active caspase 3 (https://www.rndsystems.com/products/human-mouse-active-caspase-3-antibody_af835)
Phospho-S6 ribosomal protein (https://www.cellsignal.com/products/primary-antibodies/phospho-s6-ribosomal-protein-ser235-236-antibody/2211)
CD140b (PDGFRB) (APB5) (https://www.thermofisher.com/antibody/product/14-1402-81.html?CID=AFLLO-14-1402-81)
Donkey anti-rabbit Alexa 488-conjugated (https://www.thermofisher.com/antibody/product/Donkey-anti-Rabbit-IgG-H-L-Highly-Cross-Adsorbed-Secondary-Antibody-Polyclonal/A-21206)

Donkey anti-rat Alexa 488-conjugated (https://www.thermofisher.com/antibody/product/Donkey-anti-Rat-IgG-H-L-Highly-Cross-Adsorbed-Secondary-Antibody-Polyclonal/A-21208)
Goat anti-rat Alexa 568-conjugated (https://www.thermofisher.com/antibody/product/Goat-anti-Rat-IgG-H-L-Cross-Adsorbed-Secondary-Antibody-Polyclonal/A-11077)
Donkey anti-rat Alexa 568-conjugated (https://www.abcam.com/products/secondary-antibodies/donkey-rat-igg-hl-alexa-fluor-568-preadsorbed-ab175475.html)

Anti-FoxC2 antibody is a kind gift from Dr. N Miura (Miura et al., 1997, Genomics). Previous papers from our lab have also used this antibody (Norden, P. R. et al., 2020, Elife; Tan, C. et al., 2023, EMBO Rep).

Western blotting was performed with the following antibodies:
FoxC1 (D8A6) (https://www.cellsignal.com/products/primary-antibodies/foxc1-d8a6-rabbit-mab/8758)
LAT1 (D-10) (https://www.scbt.com/p/lat1-antibody-d-10?gad_source=1&gclid=EAIaIQobChMIlbaX14yahQMV8l1HAR3CHQtHEAAYASAAEgLhrfD_BwE)
Beta-actin (https://www.ptglab.com/products/Pan-Actin-Antibody-66009-1-Ig.htm)
Goat anti-Rabbit IgG, HRP-conjugate (https://www.emdmillipore.com/US/en/product/Goat-Anti-Rabbit-IgG-Antibody-HRP-conjugate,MM_NF-12-348?ReferrerURL=https%3A%2F%2Fwww.google.com%2F&bd=1)
Donkey anti-Mouse IgG, HRP-conjugate (https://www.thermofisher.com/antibody/product/Donkey-anti-Mouse-IgG-H-L-Secondary-Antibody-Polyclonal/A16011)

Antibody used for Dynabead coating for isolation of retina endothelial cells
PECAM-1/CD31 (https://www.bdbiosciences.com/en-us/products/reagents/flow-cytometry-reagents/research-reagents/single-color-antibodies-ruo/purified-rat-anti-mouse-cd31.553370)

Antibodies used for ChIP assay:

FoxC1 (https://www.citeab.com/antibodies/731208-ab5079-anti-foxc1-antibody-chip-grade)
Control IgG (https://www.thermofisher.com/antibody/product/Goat-IgG-Isotype-Control/02-6202)

Antibodies used for assessment of purity of isolated retina endothelial cells for transcriptomic analyses

CD31 (https://www.bdbiosciences.com/en-us/products/reagents/flow-cytometry-reagents/research-reagents/single-color-antibodies-ruo/purified-na-le-rat-anti-mouse-cd31.553369)
CD45-FITC (30-F11) (https://www.biolegend.com/en-ie/products/fitc-anti-mouse-cd45-antibody-99?GroupID=BLG1932)
Alexa Fluor-647 conjugated Goat anti Rat IgG (https://www.thermofisher.com/antibody/product/Goat-anti-Rat-IgG-H-L-Cross-Adsorbed-Secondary-Antibody-Polyclonal/A-21247)

Eukaryotic cell lines

Policy information about cell lines and Sex and Gender in Research

Cell line source(s)	Human retina microvascular endothelial cells (HRMVECs) were obtained from Cell Systems (ACBRI 181)<br>Pooled human umbilical vein endothelial cells (HUVECs) were purchased from Lonza (CC-2519)
Authentication	None of the cell lines were authenticated by us.
Mycoplasma contamination	Commercially purchased cell lines were negative for mycoplasma as stated on the manufacturer's website.
Commonly misidentified lines (See ICLAC register)	No misidentified cell lines were used.

Animals and other research organisms

Policy information about studies involving animals; ARRIVE guidelines recommended for reporting animal research, and Sex and Gender in Research

Laboratory animals

Mice were housed and kept under normal lighting conditions with 12-hour-on, 12-hour-off cycles, 72 ± 2 °F temperature and 30-70% humidity range in the Center for Comparative Medicine at Northwestern University.
Endothelial cell-specific Foxc1, Foxc2, compound Foxc1, Foxc2 knockout mice and Foxc1c2/+ and Foxc1c2/c2 were generated as previously described. Foxc1c2/+ mice were crossed with one another to generate Foxc1+/+, Foxc1c2/+, and Foxc1c2/c2 mice for analysis at P6.
Analysis of Endothelial cell-specific Foxc1 knockout mice post tamoxifen treatment was carried out at postnatal day 6, 12 or 21. Mice receiving an intraperitoneal injection of MHY 1485 or DMSO were analyzed at P7. OIR related experiments and expression analysis of FOXC1/PDGFR beta/IB4 were performed at P18. Age of the animals used in each experiment has been described in the associated figure legend.
Endothelial cell-specific Foxc2, and compound Foxc1, Foxc2 knockout mice were analyzed at P6.
Slc3a2fl/+, and Slc7a5fl/+were purchased from The Jackson Laboratory.
Slc3a2fl/fl, Slc7a5fl/fl and compound Slc3a2fl/fl; Slc7a5fl/fl mice were generated by crossing Slc3a2fl/+ and Slc7a5fl/+ mice through several generations to acquire the desired genotypes.
Compound heterozygous EC-Foxc1fl/+; Slc3a2fl/+-KO, EC-Foxc1fl/+; Slc7a5fl/+-KO, and EC-Foxc1fl/+; Slc3a2fl+; Slc7a5fl/+-KO mice

were generated by crossing Cdh5-CreERT2; Foxc1fl/fl mice with Slc3a2fl/fl, Slc7a5fl/fl, or Slc3a2fl/fl; Slc7a5fl/fl mice.
EC-specific Foxc1fl/+ mice were generated by crossing wild-type mice with Cdh5-CreERT2; Foxc1fl/fl mice.
EC-specific Foxc1fl/+ mice, EC-Foxc1fl/+; Slc3a2fl/+-KO, EC-Foxc1fl/+; Slc7a5fl/+-KO, and EC-Foxc1fl/+; Slc3a2fl+; Slc7a5fl/+-KO mice were analyzed at P6.
PDGFRβ-P2A-CreERT2 mice were purchased from the Jackson Laboratory (Stock #030201) and crossed with Foxc1fl/fl mice to obtain pericyte-specific Foxc1 mutant (PDGFRβ-P2A-CreERT2; Foxc1fl/fl) mice. Pericyte-specific Foxc1 knockout mice were analyzed at P6.

Wild animals: The study did not involve use of wild animals.

Reporting on sex: Both male and female mice were used in our study.

Field-collected samples: The study did not involve animals from the field.

Ethics oversight: All animal procedures were approved by the Institutional Animal Care and Use Committee at Northwestern University.

Note that full information on the approval of the study protocol must also be provided in the manuscript.

Plants

Seed stocks: *Report on the source of all seed stocks or other plant material used. If applicable, state the seed stock centre and catalogue number. If plant specimens were collected from the field, describe the collection location, date and sampling procedures.*

Novel plant genotypes: *Describe the methods by which all novel plant genotypes were produced. This includes those generated by transgenic approaches, gene editing, chemical/radiation-based mutagenesis and hybridization. For transgenic lines, describe the transformation method, the number of independent lines analyzed and the generation upon which experiments were performed. For gene-edited lines, describe the editor used, the endogenous sequence targeted for editing, the targeting guide RNA sequence (if applicable) and how the editor was applied.*

Authentication: *Describe any authentication procedures for each seed stock used or novel genotype generated. Describe any experiments used to assess the effect of a mutation and, where applicable, how potential secondary effects (e.g. second site T-DNA insertions, mosiacism, off-target gene editing) were examined.*
